# Regulation of *HvASN1* expression by bZIP transcription factors during barley embryo development and germination

**DOI:** 10.1007/s00425-025-04730-0

**Published:** 2025-06-09

**Authors:** Estefanía Contreras, Rosario Alonso, Elena Pastor-Mora, Mar G. Ceballos, Jesús Vicente-Carbajosa, Raquel Iglesias-Fernández

**Affiliations:** 1 Centro de Biotecnología y Genómica de Plantas-Severo Ochoa (CBGP, UPM-INIA), Universidad Politécnica de Madrid (UPM) - Instituto Nacional de Investigación y Tecnología Agraria y Alimentaria (CSIC/INIA), 28223 Pozuelo de Alarcón, Spain; 2 Departamento de Biotecnología-Biología Vegetal, Escuela Técnica Superior de Ingeniería Agronómica, Alimentaria y de Biosistemas, UPM, 28040 Madrid, Spain

**Keywords:** Asparagine synthetase 1, Germination, *Hordeum vulgare*, HvBLZ1, HvbZIP53, Light regulation, Nitrogen assimilation, Nitrogen use efficiency, Seed development

## Abstract

**Main conclusion:**

Our findings provide new insights into the molecular mechanisms that regulate N metabolism in barley and potentially other cereal crops, offering valuable perspectives for enhancing N use efficiency in agricultural practices.

**Abstract:**

Efficient nitrogen (N) utilization is essential for plant growth, especially during seed development and germination. In barley (*Hordeum vulgare*), the asparagine synthetase gene *HvASN1* is essential for nitrogen transport and storage, synthesizing asparagine, a key molecule in N recycling. The phylogenetic analysis indicates that HvASN1 clusters with Arabidopsis AtASN1 and shares high similarity with HvASN2, suggesting a conserved role in N metabolism. A detailed characterization of a ~ 500 bp *HvASN1* promoter region revealed a conserved GCN-like *cis*-element. Transient expression assays in *Nicotiana benthamiana* demonstrated that the wild-type promoter significantly increases luciferase activity under dark conditions, whereas mutation of the GCN-like element reduces this activity, highlighting its role in light-responsive gene regulation. Further investigation identified the bZIP transcription factor HvbZIP53 as an activator of the *HvASN1* promoter through binding to the GCN-like element. This activation is finely tuned by sucrose via a conserved upstream open reading frame (uORF) in HvbZIP53’s 5′ UTR, which mediates sucrose-induced repression of translation. Additionally, yeast two-hybrid assays and transient expression studies in Arabidopsis provided evidence that HvbZIP53 physically interacts with HvBLZ1, a C group bZIP factor, resulting in a synergistic enhancement of *HvASN1* expression. The spatial and temporal expression analyses further revealed that *HvASN1*, *HvbZIP53*, and *HvBLZ1* are co-expressed in key seed tissues during development and germination. These findings indicate a complex regulatory network integrating environmental and metabolic signals to modulate N metabolism in barley, with implications for improving N use efficiency in cereal crops.

**Supplementary Information:**

The online version contains supplementary material available at 10.1007/s00425-025-04730-0.

## Introduction

Nitrogen (N) is an essential macronutrient for plant growth and development, playing a central role in processes such as amino acid synthesis, nucleotide metabolism, and chlorophyll production (Gaudinier et al. 2018). The plants primarily assimilate N in the form of nitrate (NO_3_^−^) and ammonium (NH_4_^+^) which are subsequently incorporated into organic compounds through key metabolic pathways (Gaufichon et al. 2010). Among N-containing metabolites, asparagine is particularly important due to its high nitrogen-to-carbon (N:C) ratio, chemical stability, and its critical role in long-distance N transport (Lam et al. 2003; Kambhampati et al. 2017). The biosynthesis of asparagine is catalyzed by asparagine synthetase (*ASN*), which amidates aspartate using glutamine or NH_4_^+^ as N donors. The regulation of *ASN* genes is highly responsive to both environmental and metabolic signals, ensuring the efficient storage and remobilization of N under diverse physiological conditions (Lam et al. 1994; Kambhampati et al. 2017). 

In *Arabidopsis thaliana*, AtASN1 is the predominant ASN isoform, playing a crucial role in N remobilization and storage (Lam et al. 1994; Gaufichon et al. 2010; Liu et al. 2022). The expression of *AtASN1* is tightly regulated by carbon availability and light conditions, being induced under carbon starvation and darkness. However, its expression is repressed in the presence of sugars such as sucrose and glucose (Fujiki et al. 2001; Baena-González et al. 2007; Dietrich et al. 2011). This regulatory pattern highlights the role of *ASN1* genes in coordinating carbon (C) and N metabolism, a crucial process for maintaining metabolic homeostasis during plant growth and seed development.

In barley (*Hordeum vulgare*), five *ASN* genes have been identified, with *HvASN1* and *HvASN2* showing the highest sequence similarity to *AtASN1* (Møller et al. 2003; Avila-Ospina et al. 2015). Similar to its Arabidopsis ortholog, *HvASN1* has been reported as a dark-inducible gene, suggesting conserved regulatory mechanisms between monocots and dicots (Raffan and Halford 2021). However, the transcriptional control of *HvASN1* is not yet fully understood, particularly in regard to the *cis-*regulatory elements and *trans-*acting factors involved in its response to environmental and metabolic signals.

Basic leucine zipper (bZIP) transcription factors (TFs) are key regulators of various physiological processes, including responses to light, metabolic signaling, and stress adaptation (Jakoby et al. 2002; Corrêa et al. 2008; Nijhawan et al. 2008; Wang et al. 2015). Within the bZIP family, the S group and C group bZIPs play key roles in nutrient sensing and seed maturation (Hanson et al. 2008; Nijhawan et al. 2008). S group bZIPs, such as AtbZIP1, AtbZIP2, and AtbZIP53, regulate genes involved in amino acid metabolism and C/N homeostasis (Dietrich et al. 2011; Krapp et al. 2011). In Arabidopsis, bZIP53 interacts with other bZIP factors to form heterodimers that bind to G-box and C-box elements in the promoters of target genes, thereby modulating their expression in response to sugar and amino acid availability (Wiese et al. 2004; Hanson et al. 2008). Furthermore, bZIP53 activity is regulated at multiple levels, including transcriptional control and translational repression through sucrose-sensitive upstream open reading frames (uORFs) in its 5'-leader sequence (Wiese et al. 2004; Rahmani et al. 2009). In cereals, the orthologs of bZIP53 have been identified, including HvbZIP53 in barley, which shares conserved DNA-binding motifs with its dicot counterparts (Corrêa et al. 2008). Since *HvASN1* is transcriptionally regulated by dark conditions and carbon availability, it is hypothesized that HvbZIP53 plays a role in its activation by direct binding to a GCN-like *cis*-element in its promoter. Additionally, C group bZIPs, such as HvBLZ1, are key regulators of seed-specific gene expression. These factors recognize GCN-like elements in the promoters of genes involved in seed maturation, including those encoding storage proteins (Vicente-Carbajosa et al. 1998; Oñate-Sánchez and Vicente-Carbajosa 2008). Furthermore, S1 and C group bZIPs can form functional heterodimers, enhancing their transcriptional activity on shared target genes (Alonso et al. 2009; Pedrotti et al. 2018). This suggests that HvbZIP53 and HvBLZ1 may cooperate in the activation of HvASN1, integrating environmental and metabolic signals to fine-tune N storage in developing barley seeds. 

This study primarily utilizes a combination of phylogenetic analysis, transient expression assays, yeast two-hybrid (Y2H) interactions, and mRNA in situ hybridization to investigate the regulatory mechanisms controlling *HvASN1* expression. By identifying conserved *cis*-regulatory elements, assessing the transcriptional activity of HvbZIP53 and HvBLZ1, and elucidating their potential interactions, this study offers a new perspective on the molecular networks regulating N assimilation and storage in barley. Specifically, *HvASN1* activation is synergistically enhanced through the collaboration of the HvbZIP53 and HvBLZ1 bZIP TFs. By gaining a deeper understanding of these regulatory pathways, we could contribute to the development of cereal crops with enhanced N use efficiency and improved seed quality, ultimately boosting agricultural productivity and sustainability.

## Materials and methods

### Plant material and growth conditions

*Hordeum vulgare* cv. Bomi (barley) seeds were surface sterilized in 1% NaOCl for 10 min and washed with sterile water. Then, they were germinated on two moistened filter papers (Whatman #3) in Petri dishes at 21 °C for 3 days in the dark. The seedlings were transferred to pots and grown in the greenhouse under long-day conditions (16 h/8 h light/darkness; light intensity 155 µmol photons m^−2^s^−1^) at 21 °C. The barley seeds were collected at different developmental stages from adult plants and classified according to their size and color: White 1 (W1; 2–3 mm), White 2 (W2; 3–4 mm), White 3 (W3; 4–6 mm), Early Green (EG; 6–8 mm) and Late Green (LG; > 8 mm).

The germination assays were performed in triplicate from 15 sterilized and stratified seeds (4 °C, 4 days), under long day conditions (16 h/8 h, light/darkness) at 21 °C. Samples were separated into embryo and endosperm at 0, 12, 24, 36, and 48 h of imbibition (hoi). The collected material was immediately frozen in liquid N and stored at − 80 °C until used for RNA extraction.

*Arabidopsis thaliana* Columbia (Col-0) seeds were sterilized (15 min in 70% ethanol followed by 20 s in 100% ethanol) before sowing in Petri dishes containing Murashige and Skoog (MS)/2 medium solidified with 0.4% Phytagel. After plating, the seeds were stratified for 3 days at 4 °C, incubated at 23 °C during 2 weeks under long-day conditions (16 h/8 h, light/darkness; light intensity 155 µmol photons m^−2^s^−1^) and then transferred to pots in the greenhouse and grown in the same conditions.

### Bioinformatic tools: HvASN1 and HvbZIP53 identification, phylogenetic dendrogram and phylogenetic shadowing

*Hordeum vulgare* ssp. *vulgare* L. *HvbZIP53*, *HvASN1, HvBLZ1*, and putative orthologous gene sequences of S group of bZIP from *Aegilops tauschii* (Aet), *Triticum aestivum* (Ta), *Triticum urartu* (Tu), *Brachypodium distachyon* (Bd), *Oryza sativa* (Os) and *Zea mays* (Zm) were retrieved from Phytozome v13 server (Supplementary Table S1; https://phytozome-next.jgi.doe.gov/).

Sequences were aligned by ClustalW tool (Thompson et al. 1994) and phylogenetic dendrogram was constructed using MEGA 4.1 software (Kumar et al. 2008), with the neighbor-joining algorithm, a bootstrap analysis with 1000 replicates, complete deletion of alignment gaps, and Jones–Taylor–Thornton matrix as settings. To identify motifs in these sequences and to validate phylogenetic trees, the MEME tool version 5.0.4 was employed with default parameters except that the maximum number of motifs to find was set from 5 to 15 and the minimum width was set to 10 amino acid residues (Bailey et al. 2009; https://meme-suite.org/tools/meme). The consensus sequences in Table S2 and Supplementary Fig. S3 indicate the amino acids appearing in a given position with a probability > 0.2. When more than one amino acid satisfies that condition for one particular position, these amino acids are indicated between square brackets.

The *ASN1* promoter sequences (*Aegilops tauschii*, *Hordeum vulgare* ssp. *vulgare* L., *Triticum aestivum*, *Triticum urartu*) were obtained from Phytozome v13 server (Supplementary Table S3) and analyzed by the m-Vista Shuffle LAGAN web toolkit that was used to create pairwise alignments of these promoters (Frazer et al. 2004; https://genome.lbl.gov/vista). The sequences of conserved regions within promoters (phylogenetic shadowing) were analyzed with T-coffee (Notredame et al. 2000; https://tcoffee.crg.eu/apps/tcoffee). Plant *cis*-acting regulatory DNA elements were searched through the following databases: PLACE with a cutoff of > 4 bp (Higo et al. 1999; https://www.dna.affrc.go.jp/PLACE), MotifFinder (https://www.genome.jp/tools/motif) and PlantCare (Lescot et al. 2002; https://bioinformatics.psb.ugent.be/webtools/plantcare/html).

### Total RNA isolation and real-time quantitative PCR analyses

Total RNA was purified from roots, leaves, flowers and stems (12-week-old plants), from seeds at different stages of development (W1, W2, W3, EG, and LG) and at different time points of germination (0, 12, 24, 36, and 48 hoi) as described (Oñate-Sánchez and Vicente-Carbajosa 2008). RNA samples were treated with DNAse I, RNAse-free (Roche) to remove genomic DNA contamination.

cDNA was first synthesized from 1 µg of total RNA using the First-Strand Synthesis kit for RT- (Roche) following manufacturer’s instructions. Samples were stored at − 20 °C until used for amplification with specific primers (Supplementary Table S4). The expression of glyceraldehyde 3-phosphate dehydrogenase (*HvGAPDH*, HORVU7Hr1G074690) gene was used to normalize the data, since it was demonstrated to be constant throughout the studied period (ΔC_T_, Supplementary Fig. S6). Real-time quantitative PCR (qPCR) analyses were performed in an Eco^®^ Real-Time PCR System (Illumina; https://www.illumina.com). For each 10 µl of reaction, 1 µl cDNA sample was mixed with 5 µl of FastStart Universal SYBR Green Master (ROX; Roche), 0.25 µl of each primer (final concentration 200 nM), plus sterile water up to the final volume. Samples were subjected to thermal-cycling conditions of 95 °C for 10 min and 40 cycles of 10 s at 95 °C followed by 30 s at 60 °C of annealing and extension. The melting curve was designed to increase from 55 °C to 95 °C; melting temperatures for each amplicon and primer efficiency, estimated via a calibration dilution curve and slope calculation, is shown in Supplementary Table S4. The technical duplicates of three biological replicates for each time point were analyzed. Expression levels were determined as the number of cycles needed for the amplification to reach a threshold level fixed in the exponential phase of the PCR reaction (C_T_; Pfaffl 2001).

### Yeast two-hybrid assays

pGAD424 and pGBT9 effector plasmids (Clontech), containing the alcohol dehydrogenase I (*AdhI*) promoter fused to the Gal4 DNA-activation domain (*Gal4AD;* pGAD424 prey vector) and the Gal4 DNA-binding domain (*Gal4BD;* pGBT9 bait vector) were used to generate translational fusions with *HvBLZ1* (*HvBLZ1* cDNA from *H. vulgare* cv. Bomi has been previously characterized in our lab (Vicente-Carbajosa et al. 1998) and *HvbZIP53* coding sequence (CDS).

*Saccharomyces cerevisiae* haploid strain SFY526 (Clontech) carrying the *LacZ* (β-galactosidase) reporter gene under the control of a truncated *Gal1* promoter that contains Gal4-responsive elements (*Gal1-UAS*) was transformed, and β-galactosidase activity was quantified as described in Vicente-Carbajosa et al. (1998).

### mRNA in situ hybridization experiments

The protocol used is a modification of that described in Ferrándiz et al. (2000). Barley developing (W1 and LG stages) and germinating (30 hoi) seeds were collected and immediately fixed in the FAE solution (formaldehyde: acetic acid: ethanol: water; 3.5:5:50:41.5, by vol.) for 45 min under mild vacuum, and left for 6 days with gentle shaking at 4 °C. The samples were dehydrated through a graded ethanol series, then embedded in paraffin, sectioned to 10 µm and de-waxed. Prior to the hybridization protocol, sections were stained with 0.05% (*w*/*v*) Toluidine Blue (Merck) to verify tissue integrity. A pre-hybridization treatment was performed by incubating the sections in 0.2 M HCl, neutralizing, and digesting with 1 mg/ml proteinase-K. Then tissue sections were again dehydrated in ethanol dilution series, before applying the hybridization solution (100 mg/ml tRNA, 6X saline-sodium citrate buffer (SSC), 3% formamide) containing approximately 100 ng/ml antisense or sense digoxigenin-labeled RNA probes, corresponding to DNA fragments (200–300 bp) derived from the 3’-non coding regions of barley *HvbZIP53* and *HvASN1* genes (Supplementary Table S4), synthesized with DIG RNA labeling mix (Roche) according to the manufacturer’s specifications. Hybridization was performed overnight at 52 °C followed by two washes in 2X SSC and 50% formamide for 90 min at the same temperature. Antibody incubation and color detection were carried out according to the manufacturer’s instructions. The sections were examined under a Zeiss Axiophot Microscope (Zeiss) and images were captured and processed with the Leica Application Suite 2.8.1 software (Leica).

### Transient expression assays

For transient expression in *Nicotiana benthamiana* leaves, *HvASN1* promoter (*P*_*HvASN1*_, 803 bp; Supplementary Table S3) was amplified by PCR from barley genomic DNA using specific primers (Supplementary Table S4) and subcloned into the *Eco*RI-*Xho*I sites in the pENTR3C vector. *P*_*HvASN1-mut*_ (802 bp) was created by PCR amplification of *P*_*HvASN1*_*,* using specific primers containing the mutations (Supplementary Table S4). Then, the sequences were transcriptionally fused to the luciferase reporter gene (*LUC*) into the plasmid pGWB435 (Nakagawa et al. 2007) by Gateway^™^ cloning system (Invitrogen, Thermo Fisher Scientific). *HvbZIP53* CDS was amplified from *H. vulgare* cv. Bomi cDNA using the primers in Supplementary Table S4 and cloned into the pROKII vector (Baulcombe et al. 1986) under the control of the cauliflower mosaic virus (CaMV) 35S promoter. The constructs were introduced into *Agrobacterium tumefaciens* GV3101 strain by electroporation. *N. benthamiana* leaves were infiltrated with bacterial suspensions containing the constructs and those containing the plasmid *pBIN61::35 s::P19* to avoid genome silencing (Voinnet et al. 2003). Quantitative LUC enzymatic activity was assayed according to Lasierra and Prat (2018). For statistical analysis of quantitative LUC activity data, a Student’s t-test was performed.

For transient expression assays in *A. thaliana*, *P*_*HvASN1*_*::uidA* reporter was constructed by fusing *P*_*HvASN1*_ (541 bp) to the ß-glucuronidase (*GUS)* reporter gene (Mena et al. 1998). *P*_*35S*_*::HvBLZ1* (Vicente-Carbajosa et al. 1998), *P*_*35S*_*::HvbZIP53* and *P*_*35S*_*::HvbZIP53-uORF* constructs were prepared by cloning the corresponding CDS in the pBlueScript vector (Stratagene) under the control of CaMV 35S promoter followed by the first intron of the maize *AdhI* gene, and then subcloning at the *Bam*HI*-Xho*I sites of this plasmid (Mena et al. 1998). *P*_*35S*_*::HvbZIP53-uORF* construct was subsequently cloned into the pGreen plasmid (Hellens et al. 2000) containing the neuraminidase (*NAN*) gene (Kirby and Katanagh 2002). The transformation efficiency was normalized to GUS enzymatic activity (Kirby and Katanagh 2002). Particle bombardment was carried out on 5 *A. thaliana* leaves with a biolistic helium gun device (DuPont PSD-1000, Bio-Rad Laboratories) as described (Abraham et al. 2016). The leaves were then incubated for 24 h in MS/2 medium in the absence and in the presence of sucrose (0, 20, 50, and 100 mM) and subsequent GUS and NAN enzymatic activities were evaluated as previously described (Alonso et al. 2009).

## Results

### *HvASN1* gene promoter contains an evolutionary conserved GCN-like element responding to dark

AtASN1 is suggested to have a role in N transport and storage in seeds. It is induced under dark conditions and repressed in the presence of carbon sources (Lam et al. 1994). In barley, five *ASN* genes have been identified from the genome data. Among them, *HvASN1* and *HvASN2* belong to class I and cluster with AtASN1, suggesting they may share a similar function. Interestingly, *HvASN1* has been also described as a dark-inducible gene. *HvASN1* and *HvASN2* show a high sequence identity (80.1%), which has made difficult to distinguish both transcript types (Møller et al. 2003; Avila-Ospina et al. 2015; Raffan and Halford 2021).

An unrooted phylogenetic tree has been constructed using 1 and 2 group ASN protein sequences from *H. vulgare* and related Triticeae species, including *Aegilops tauschii, Triticum aestivum,* and *Triticum urartu*. As an outgroup, *A. thaliana* AtASN1 protein sequence was used (Fig. S1A and Supplementary Table S1). All sequences share the majority of motifs, including those containing amino acids critical for glutamine amido-transferase (GATase) and ASN activities. The exception is motif 19, which is found only in Triticeae ASN1 proteins (Fig. S1B, Table S2).

**Fig. 1:**
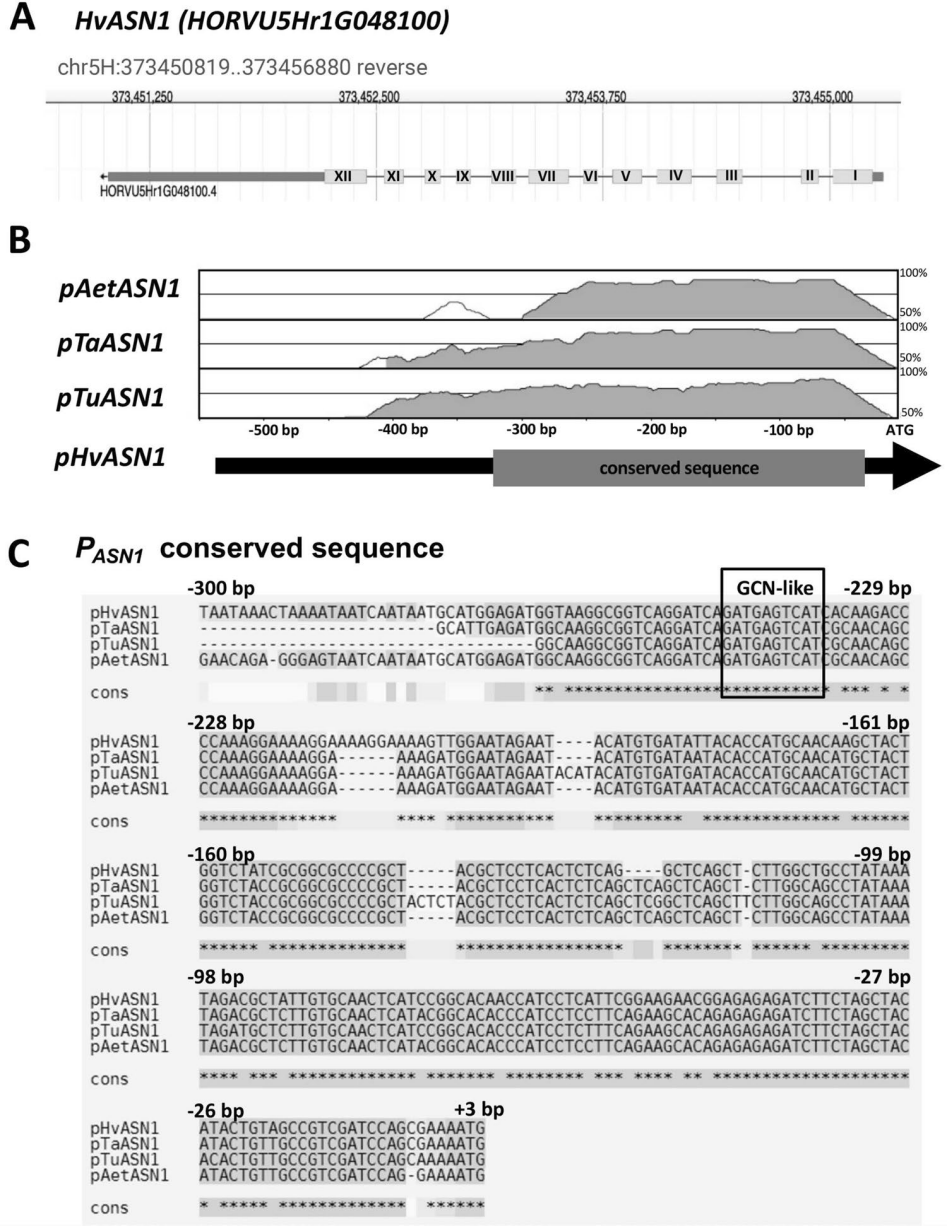
Identification of a conserved GCN-like *cis*-element in the Triticeae *ASN1* promoters. **A** Genome position and browser view of the *HvASN1* locus on chromosome 5 in *Hordeum vulgare* ssp. *vulgare* L. Exons of *HvASN1* are represented as gray boxes, and introns as lines. The arrow indicates the direction of transcription. **B** Pairwise alignment of *ASN1* promoter sequences from different Triticeae species, using phylogenomic analysis with the mVISTA tool. Graphical output shows base pair % identity in a sliding window of 100 bp with a 70% identity threshold. Gray areas indicate a conserved block. **C** Sequence alignment of the conserved block identified in **B** in the Triticeae *ASN1* promoters. The GCN‐like box is indicated. *Aet, Aegilops tauschii*; *Ta, Triticum aestivum*; *Tu, Triticum urartu; Hv, Hordeum vulgare*

To identify genomic regions involved in the transcriptional control of *HvASN1*, the putative promoter region of this gene (~ 500 bp upstream of the start codon; Fig. 1A) has been searched for conserved *cis*-elements based on its homology to the corresponding region in the other four Triticeae orthologs (Fig. 1 and Supplementary Table S3). Pairwise alignment of the promoter sequences from the four orthologous *ASN1* genes reveals a highly conserved block spanning from − 266 bp to the start codon (314 bp; Fig. 1B) which includes a GCN-like bZIP binding site (5′-GATGAGTCAT-3′) located between 247 and 237 bp upstream of the start codon (Fig. 1C). It has been described that the GCN-like motif, together with the prolamin box, constitutes the endosperm box, which plays a key role in activating the expression of seed storage protein genes in the endosperm of cereals (Vicente-Carbajosa et al. 1998).

**Fig. 2:**
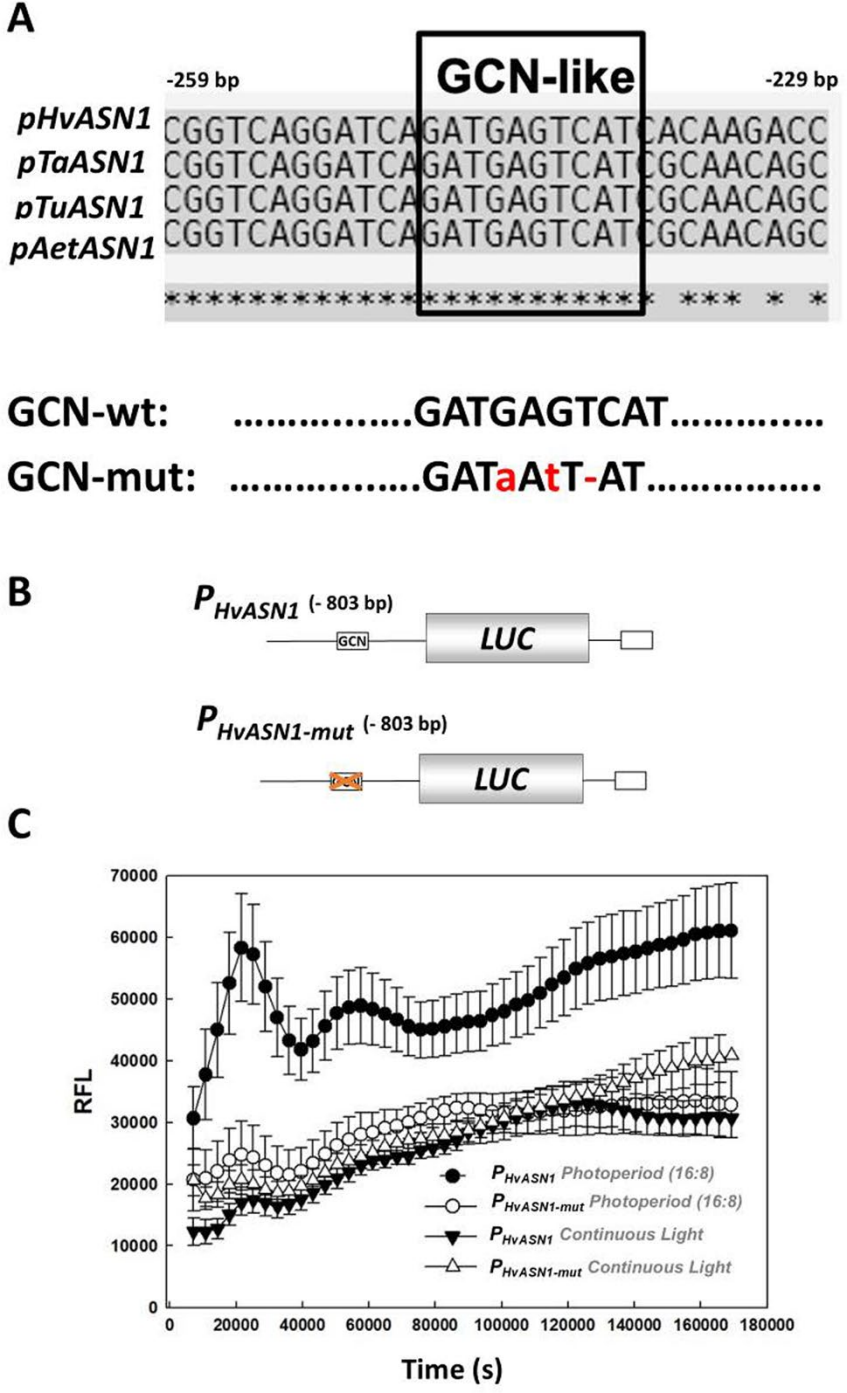
The *HvASN1* promoter responds to darkness. Transient expression assays of luciferase enzymatic activity driven by the promoter of *HvASN1* gene (*P*_*HvASN1*_), and by a mutated version (*P*_*HvASN1-mut*_) in *Nicotiana benthamiana* leaves. **A** Alignment of sequences spanning − 259 to − 229 bp from the initial codon within the conserved block identified in Fig. 1 from different Triticeae species. A conserved GCN-like sequence is highlighted in a square, and a mutated version with 2-bp changes and a 1-bp deletion in the GCN-like box (GCN-mut) is shown. **B** Schematic representation of the two reporter constructs used in transient expression assays. **C** Transient expression assays in *Nicotiana benthamiana* leaves with the two constructs described in **B**, in response to different light conditions: photoperiod (16 h:8 h; light:dark) and continuous light. Values are means ± standard error (SE) of eight independent replicates. Differences between *P*_*HvASN1*_*::LUC* and *P*_*HvASN1-mut*_*::LUC* at all timepoints measured are not significant under photoperiod conditions, and significant under continuous light, except for the first timepoint (Student’s t-test, *P* ≤ 0.05)

To explore the functional relevance of the GCN-like element in the *HvASN1* promoter (*P*_*HvASN1*_), constructs of the full *P*_*HvASN1*_, which contains a wild type version of the GCN-like element, and *P*_*HvASN1-mut*_, which contains a mutated version (5´-GAT**a**A**t**T**-**AT-3´; Fig. 2A) have been generated and fused to the luciferase reporter gene (*LUC*; Fig. 2B). The constructs *P*_*HvASN1*_*::LUC* (803 bp) and *P*_*HvASN1-mut*_*::LUC* (802 bp) have been used for transient expression assays via agro-infiltration in *Nicotiana benthamiana* leaves. Since the *HvASN1* gene is known to be a dark-inducible gene, the transient expression assays were conducted under a photoperiod (16 h/8 h, light/darkness), with continuous light conditions used as a control (Fig. 2C; Fig. S2). As expected, luciferase activity controlled by the wild-type promoter under photoperiod conditions is six-fold higher than that observed under continuous light, suggesting that its expression is activated by darkness and maintained during the subsequent light exposure. Interestingly, a reduction in luciferase activity is observed under both photoperiod and continuous light conditions when the GCN-like element is mutated (Fig. 2C). These results indicate that the GCN-like element within the *HvASN1* promoter plays a crucial role in the gene's response to dark conditions. Its mutation leads to a significant reduction in promoter activity, suggesting that this element is essential for the proper transcriptional activation of *HvASN1* in response to darkness and its sustained expression upon light exposure.

### HvbZIP53 activates the *HvASN1* promoter through the GCN-like element *in planta*

In Arabidopsis, several TFs from the S group have been implicated in the regulation of *AtASN1* by binding to a G-box *cis*-element in *AtASN1* promoter. Arabidopsis S group bZIPs, along with the orthologous bZIP53 in *H. vulgare* and Poaceae species, share a motif that contains key amino acids in the conserved basic region, which mediates sequence-specific DNA binding in bZIPs (motif 1; Fig. S3).

**Fig. 3:**
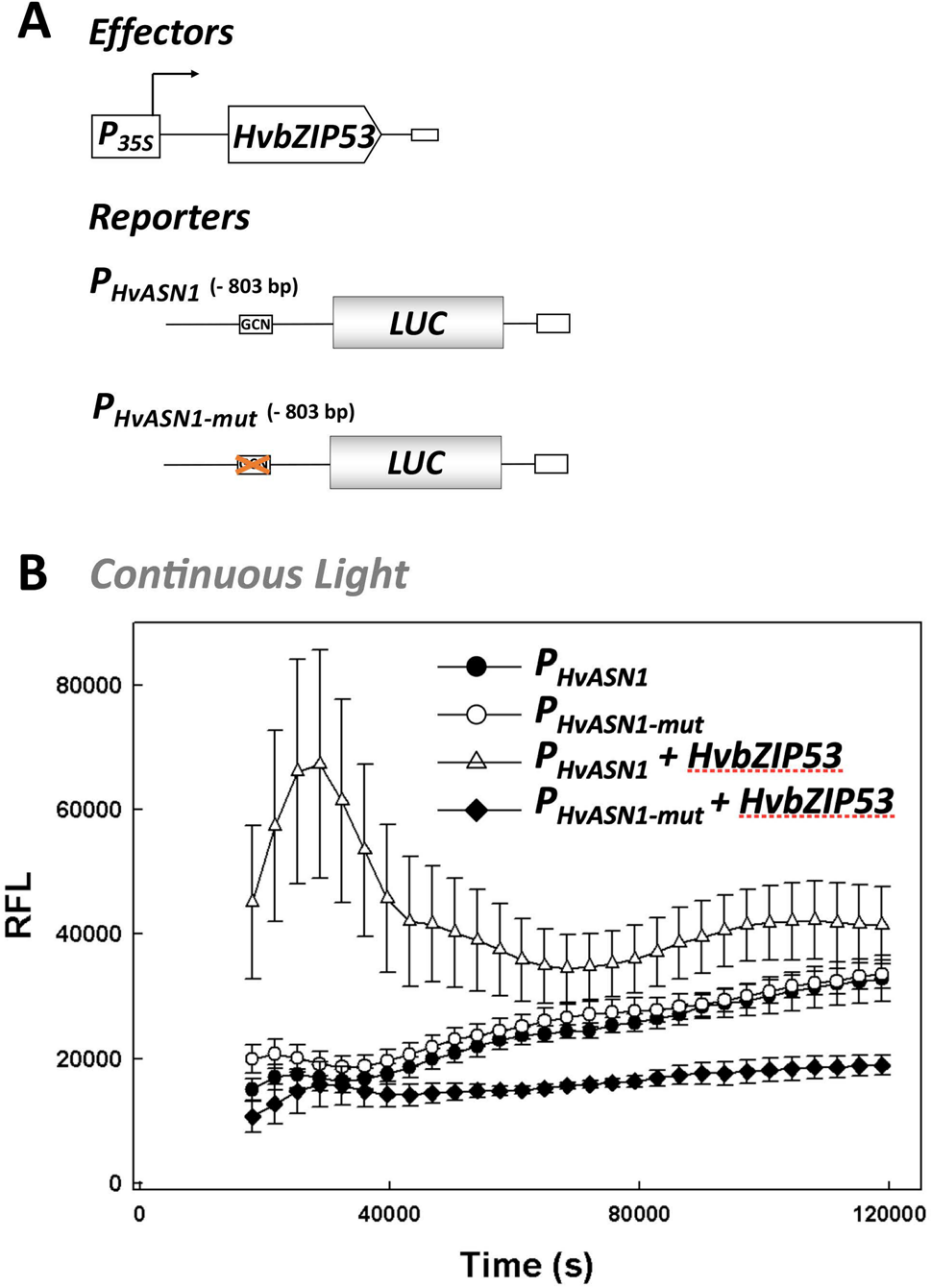
The *HvASN1* promoter is activated by HvbZIP53. Transactivation assays using as an effector the HvbZIP53 CDS under the transcriptional control of the CaMV 35S*,* and as reporter the expression of the LUC activity driven by the *HvASN1* gene promoter (*P*_*HvASN1*_) and its mutated version *(P*_*HvASN1-mut*_*)*. **A** Schematic representation of the effector and reporter constructs used in the assay. **B** Transactivation assays in *N. benthamiana* leaves under continuous light using the effector and reporter construct depicted in **A**. The relative amounts of reporter and effector plasmids used in these assays correspond to 1:1 ratio. Values are means ± SE of eight independent replicates. Differences between *P*_*HvASN1*_*::LUC* and *P*_*HvASN1-mut*_*::LUC* at all timepoints measured are not significant except for the first timepoint, and differences between *P*_*HvASN1*_*::LUC* + *HvbZIP53* and *P*_*HvASN1-mut*_*::LUC* + *HvbZIP53* are significant at all timepoints (Student’s t-test, *P* ≤ 0.05)

To determine whether the *HvASN1* gene is regulated by HvbZIP53 TF through the GCN-like *cis*-element, the interaction between HvbZIP53 and the GCN-like element has been investigated *in planta*, using a tobacco transient expression system. *P*_*HvASN1*_*::LUC* and *P*_*HvASN1-mut*_*::LUC* have been used as reporter constructs, while the *P*_*35S*_*::HvbZIP53* construct containing *HvbZIP53* gene under the control of the CaMV 35S promoter, served as the effector (Fig. 3A). The luciferase activity has been measured in leaves of *Nicotiana benthamiana* co-infiltrated with *Agrobacterium tumefaciens* carrying one of the reporter constructs and either the effector or the corresponding empty vector as a negative control. For at least three independently transformed plants, in continuous light conditions—when *HvASN1* promoter is not induced—co-transformation of the P_HvASN1_::*LUC* reporter construct with the P_35S_::*HvbZIP53* effector resulted in a sixfold the LUC activity in comparison to the reporter alone (Fig. 3B). However, when the mutated *P*_*HvASN1-mut*_*::LUC* reporter construct was co-infiltrated with *P*_*35S*_*::HvbZIP53*, LUC activity does not increase compared with *P*_*HvASN1-mut*_*::LUC* reporter alone, and even showing an slight decrease (Fig. 3B). These findings revealed the importance of the GCN-like element in *HvASN1* regulation mediated by HvbZIP53.

### *HvbZIP53* expression is fine-tuned by sucrose at post-transcriptional level

Post-transcriptional control of S1 group of bZIP TFs by sucrose binding to a uORF has been described in *A. thaliana* (Wiese et al. 2004).

**Fig. 4:**
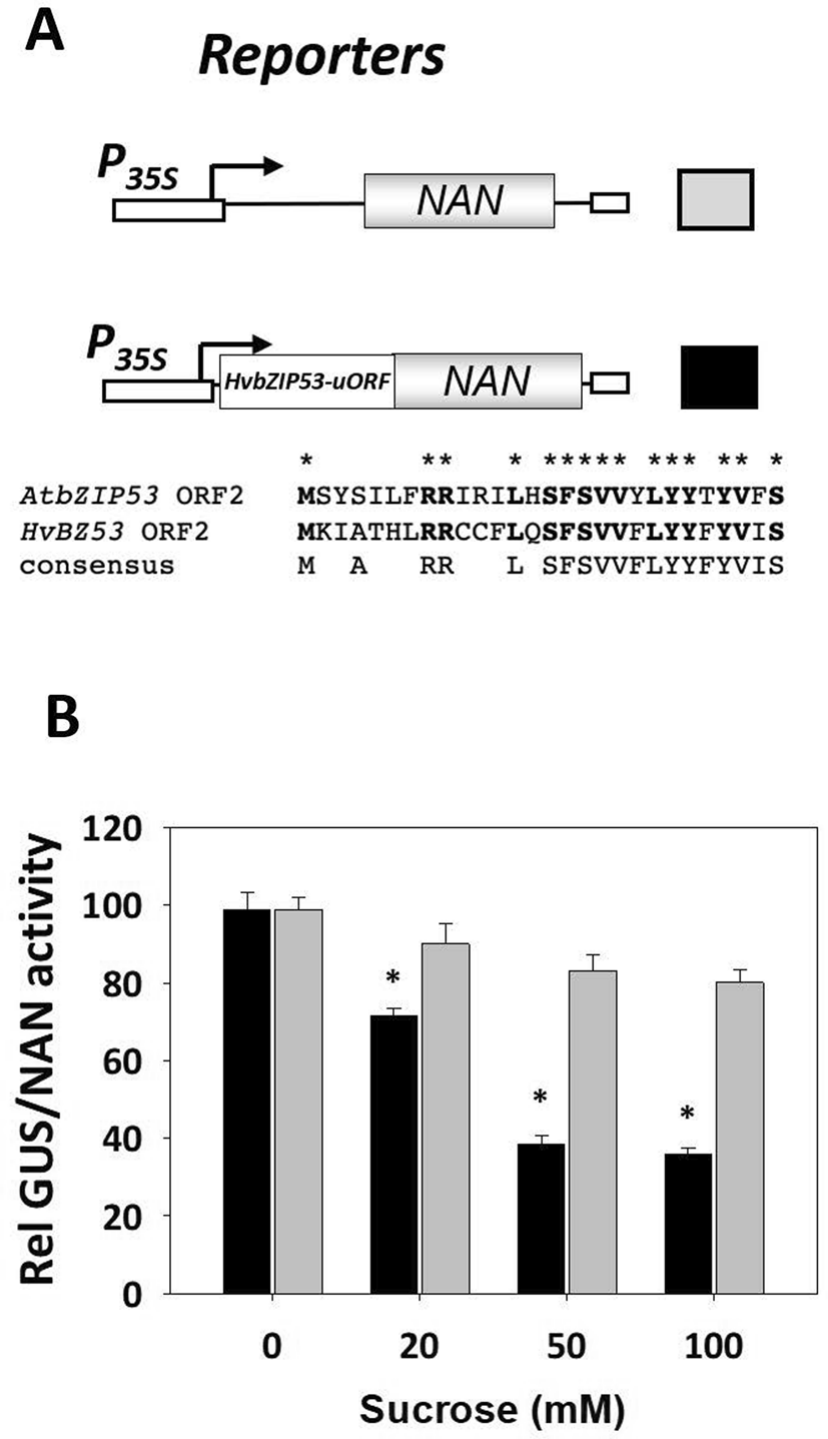
The HvbZIP53 abundance is controlled by carbon availability. **A** Schematic representation of reporter constructs used in transactivation assays. In the control construct, the *NAN* reporter is expressed under the control of the constitutive CaMV 35S promoter*.* In the second construct, the upstream ORF of the TF HvbZIP53 *(HvbZIP53-uORF)* is translationally fused to the *NAN* reporter in the N-terminus. *HvbZIP53* and *AtbZIP53-uORF* sequences are compared at the bottom. **B** Transient assays of the reporters described in **A** in bombarded *A. thaliana* leaves in the absence and in the presence of sucrose (0, 20, 50, 100 mM; 24 h of incubation). The GUS/NAN activity ratio has been used to standardize the variations in the efficiency of the transformation of the assay. Values are means ± SE of two independent assays. Asterisks (*) indicate significant differences between *HvbZIP53* and *AtbZIP53-uORF* constructs (Student’s *t*-test, *P* ≤ 0.05)

As a member of the S1 group, the *HvbZIP53* transcript also contains a conserved uORF (Fig. 4A). To investigate its function, a construct of HvbZIP53 uORF fused to the neuraminidase (*NAN*) reporter gene and driven by the 35S promoter (*P*_*35S*_*::HvbZIP53-uORF::NAN*) has been used in transient expression assays in Arabidopsis leaves, both in the absence and presence of sucrose (20, 50, and 100 mM; Fig. 4A). As shown in Fig. 4B, bombardment of Arabidopsis leaves with the *P*_*35S*_*::HvbZIP53-uORF::NAN* construct in the presence of increasing sucrose concentrations resulted in a decrease in *NAN* activity compared to the control construct (*P*_*35S*_*::NAN*). Taken together, our results confirm the role of the HvbZIP53-uORF as a transcriptional regulator of the bZIP53 transcript, mediated by sucrose.

### HvBLZ1 interacts and cooperates with HvbZIP53 in *HvASN1* activation

**Fig. 5:**
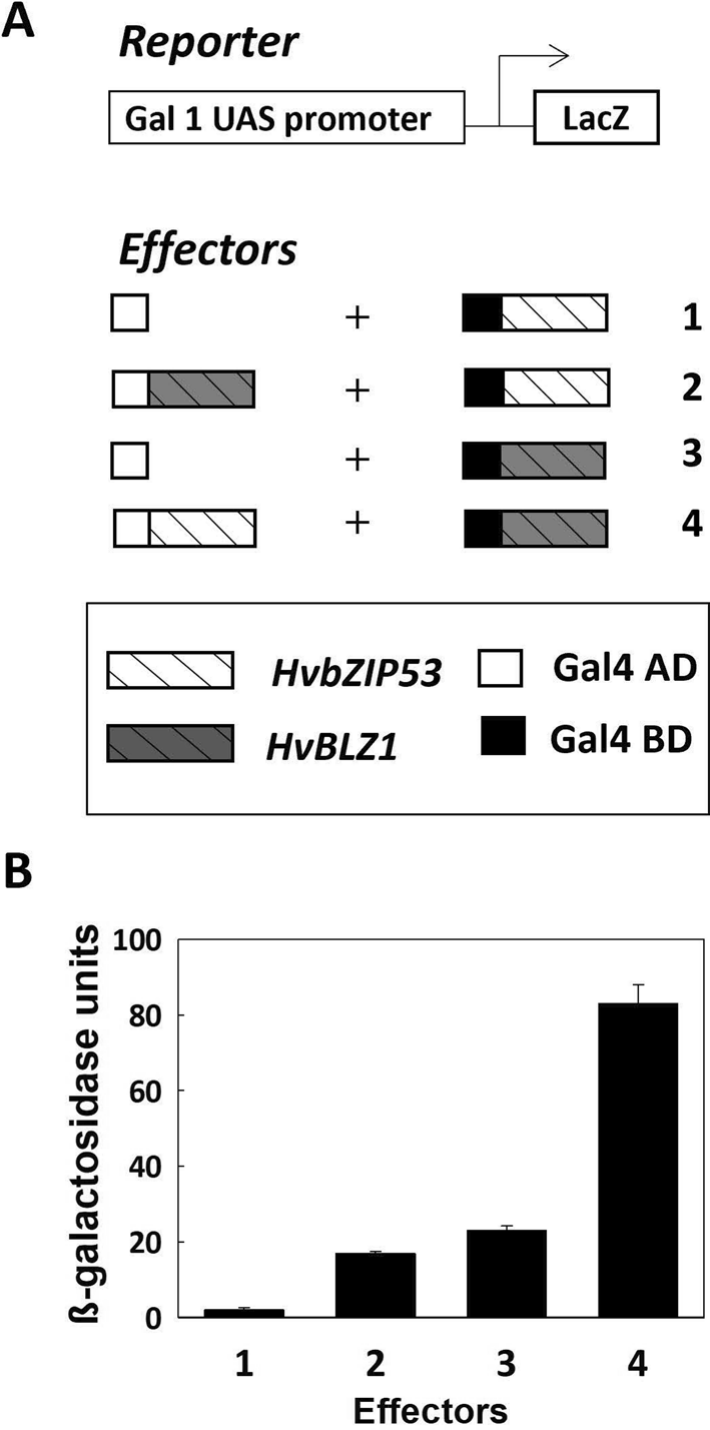
Protein–protein interaction of HvbZIP53 and HvBLZ1 TFs by Y2H. **A** Schematic representation of reporter and effector constructs used for the Y2H assays: The *Gal1-UAS* promoter is transcriptionally fused to the *LacZ* reporter gene*; HvbZIP53* or *HvBLZ1* (complete CDS) are expressed as translational fusions to the Gal4 DNA-binding domain (BD) or to the activation domain (AD). **B** Quantification of β-galactosidase activity in the Y2H assays using the effector combinations described in **A**

It has been described that the GCN-like motif is recognized by HvBLZ1, a C group bZIPs TF that acts as an activator of a seed prolamin genes (Vicente-Carbajosa et al. 1998). Since S and C group bZIPs are known to form functional heterodimers, both HvbZIP53 and HvBLZ1 recognize the GCN-like motif and are involved in the activation of seed maturation genes. To investigate the potential protein–protein interaction between HvbZIP53 and HvBLZ1, Y2H assays have been performed. The CDS of both genes have been translationally fused to the yeast Gal4 activation domain (AD) or binding domain (BD) to create effector constructs (Fig. 5A). Yeast cells transformed with the *Gal4BD-HvbZIP53* construct show detectable background levels of *LacZ* reporter activity, which increases when the strain is co-transformed with the *Gal4AD-HvBLZ1* construct. Similarly, yeast cells co-transformed with the *Gal4BD-HvBLZ1* and *Gal4AD-HvbZIP53* constructs show an increase of *LacZ* reporter compared to the control cells transformed with *Gal4BD-HvBLZ1* alone (Fig. 5B). These results indicate that HvbZIP53 interact with HvBLZ1.

The cooperative role of both TF in enhancing the transcription of *HvASN1* has been investigated. The transient expression assays in Arabidopsis leaves have been performed using the HvbZIP53 and HvBLZ1 coding sequences under the 35S promoter as effector constructs, and the *HvASN1* promoter (− 314 bp upstream of the ATG initiation codon) fused to the *GUS* gene as a reporter (*P*_*HvASN1*_*::uidA*) (Fig. S4A). In this system, co-bombardment of Arabidopsis leaves with either the *HvbZIP53* or *HvBLZ1* constructs and the *P*_*HvASN1*_*::uidA* reporter does not alter GUS activity compared to the reporter construct alone. However, when both effector constructs are co-bombarded with the reporter, there is a positive trans-activation that is three times higher than that of *P*_*HvASN1*_*::uidA* construct alone (Fig. S4B), suggesting that the presence of both *HvbZIP53* and *HvBLZ1* produces a synergistic effect on *HvASN1* activation.

Taken together, these findings indicate that HvbZIP53 and HvBLZ1 not only physically interact but also functionally cooperate to enhance the transcriptional activation of *HvASN1*. This suggests that *HvASN1* regulation in seeds may rely on the combined action of these transcription factors, highlighting the relevance of S and C group bZIP interactions in modulating gene expression during barley seed maturation and germination.

### *HvASN1*,* HvbZIP53* and *HvBLZ1* are co-expressed in the same tissues during barley seed development and germination

**Fig. 6:**
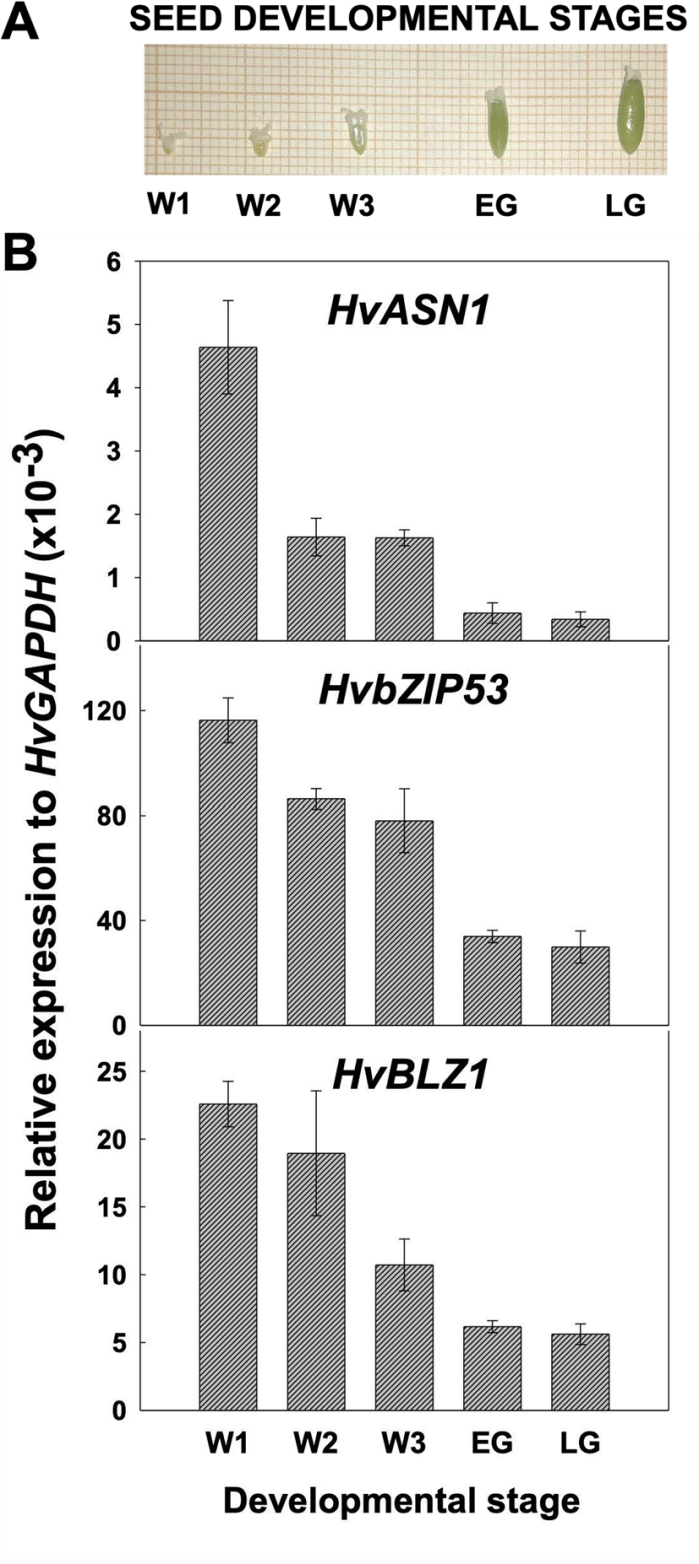
Expression of *HvASN1*, *HvbZIP53*, and *HvBLZ1* in different stages of *Hordeum vulgare* seed development.** A** Photograph of *H. vulgare* seeds in White 1 (W1), White 2 (W2), White 3 (W3), Early Green (EG), and Late Green (LG) seed developmental stages. **B** Expression analysis of *HvASN1* and *HvbZIP53* genes by qPCR throughout barley seed development. Data are means ± SE of two technical replicates from three biological samples

To investigate the spatiotemporal expression pattern of barley *HvASN1, HvbZIP53* and *HvBLZ1*, transcript accumulation was examined by qPCR in different organs (flowers, leaves, roots and stems) of 2-week-old plants (Fig. S5), as well as at different stages of seed development: White 1 (W1; 2–3 mm), White 2 (W2; 3–4 mm), White 3 (W3; 4–6 mm), Early Green (EG; 6–8 mm), and Late Green (LG; > 8 mm) (Fig. 6A), with GAPDH used as a reference gene (Fig. S6). *HvASN1* expression is almost exclusively detected floral organs of the plants, whereas *HvbZIP53* and *HvBLZ1* show widespread expression in all the tissues analyzed (Fig. S5). In developing seeds, all three genes exhibit the highest expression at the first stage of development (W1), with expression levels progressively decreasing until LG stage (Fig. 6B).

**Fig. 7:**
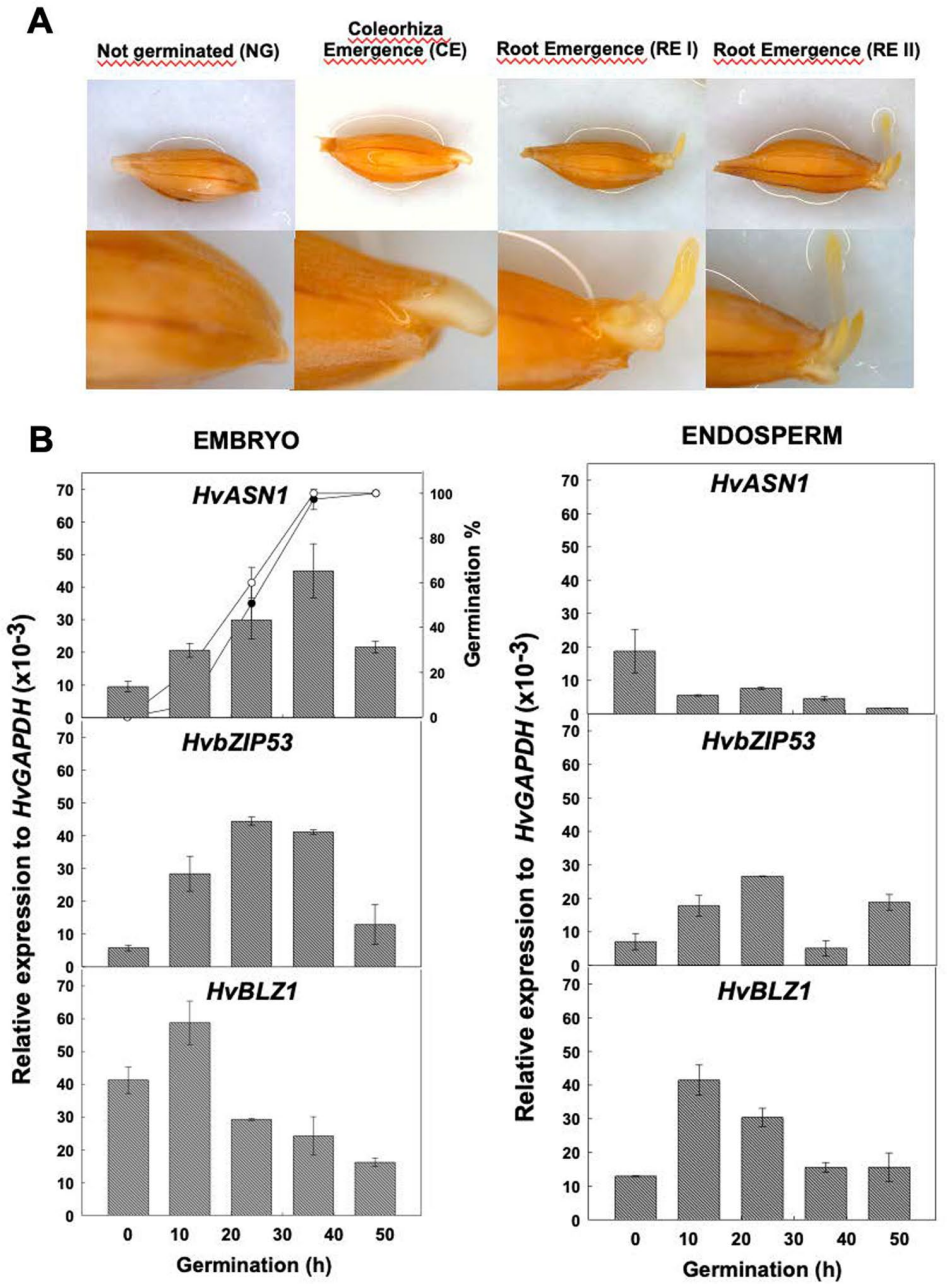
Expression of *HvASN1*, *HvbZIP53*, and *HvBLZ1* upon *Hordeum vulgare* seed germination.** A** *Hordeum vulgare* seeds in different stages of germination (non-germinated, coleorhiza emergence, root emergence stage I and II). **B** Transcript accumulation of *HvASN1, HvbZIP53,* and *HvBLZ1* genes during barley seed germination (0, 12, 24, 36, 48 h of imbibition). The time required to reach 50% of coleorhiza (CE; ○) and root emergence (RE; ●) is indicated. Data are means ± SE of two technical replicates of three biological samples

The time course of sensu stricto germination of *H. vulgare* seeds occur in two steps: first the coleorhiza emergence (CE), followed by root emergence (RE) (Fig. 7A). The expression analyses of *HvASN1, HvbZIP53*, and *HvBLZ1* have been performed in dissected embryos and endosperm at different time points during barley seed germination. In the embryo, *HvASN1* and *HvbZIP53* transcripts levels increase as germination proceeds, peaking at 36 and 24 hoi, respectively, just before reaching 50% germination sensu stricto (t_50_ CE = 21 h; t_50_ RE = 25 h), after which expression decreases until 48 hoi (Fig. 7B). *HvBLZ1* transcript accumulation peaks at 12 hoi, then progressively decreases. In de-embryonated seeds (endosperm), *HvASN1* expression is much lower during seed imbibition compared to dry endosperms. In contrast, *HvbZIP53* and *HvBLZ1* show a different expression patterns, with mRNA levels peaking at 24 hoi and 12 hoi, respectively (Fig. 7B). Our expression data show a similar temporal expression profile for both genes during seed development and in the embryo during germination.

**Fig. 8:**
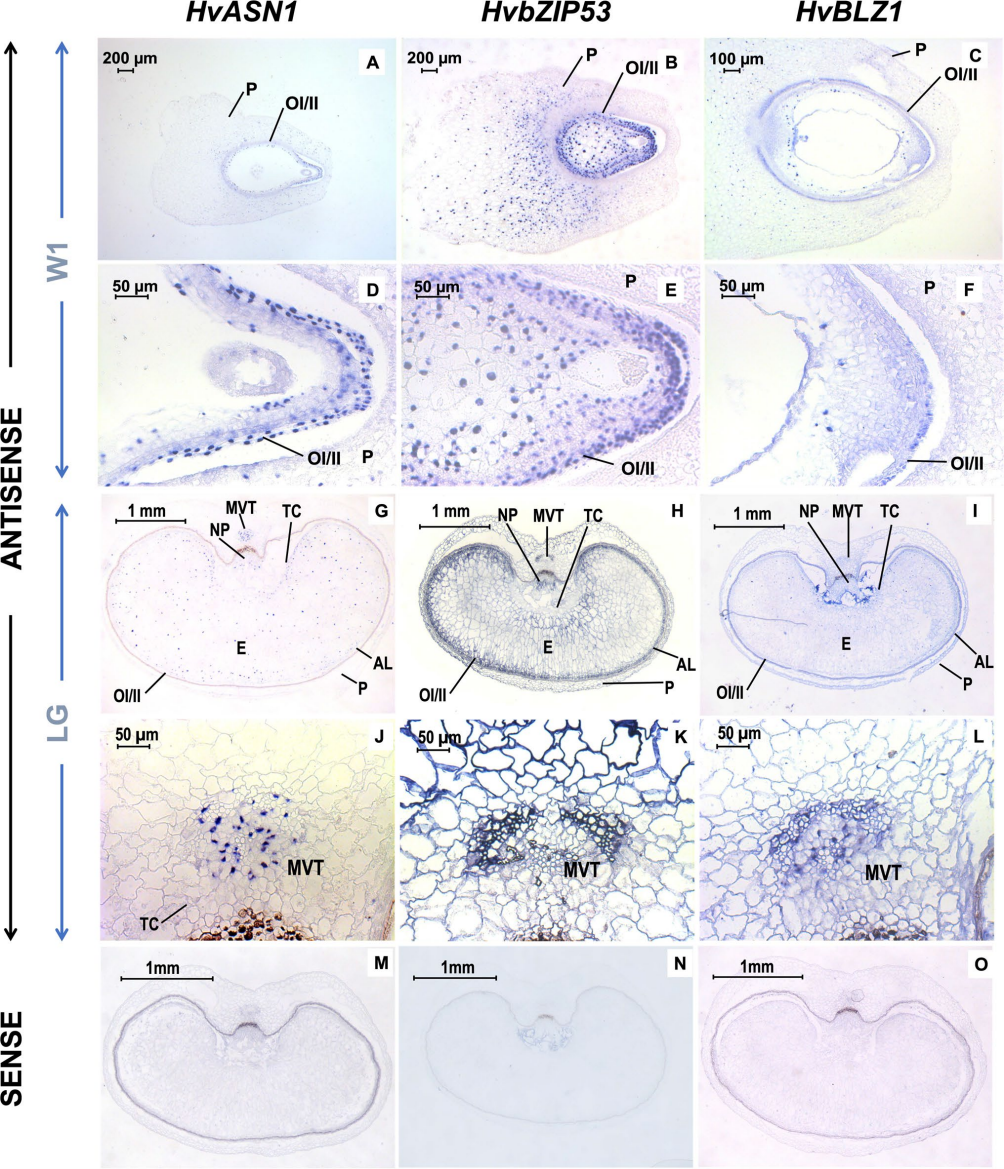
In situ mRNA hybridization analysis of *HvASN1, HvbZIP53*, and *HvBLZ1* in developing barley seeds. **A**, **D**, **G**, **J**, **M** *HvASN1*. **B**, **E**, **H**, **K**, **N** *HvbZIP53.* **C**, **F**, **I**, **L**, **O** *HvBLZ1.* **A**–**C** Longitudinal sections of White 1 stage (W1) seeds. **D**–**F** Close-up of the seed basal zone. **G**–**I** Crosswise sections of Late green stage (LG) grains. **J**–**L** Close-up of the main vascular tissue. **M**–**O** Control sense probes. AL, aleurone layer; E, endosperm; MVT, main vascular tissue; NP, nucellar projection; OI/II, outer integument/inner integument; P, pericarp; TC, transfer cells

To determine whether *HvASN1, HvbZIP53* and *HvBLZ1* transcripts co-localize in developing seeds, *mRNA *in situ hybridization assays have been performed at the W1 and LG stages (Fig. 8A–L), using specific antisense probes derived from the 3´ non-coding regions of these genes (Supplementary Table S4). In longitudinal sections at the W1 stage, a strong signal for *HvASN1, HvbZIP53 and HvBLZ1* transcripts is detected in the Outer and the Inner Integuments (OI/II) of the embryo sac (Fig. 8A–F). In both transversal and longitudinal sections at the LG stage, the *HvASN1, HvbZIP53* and *HvBLZ1* mRNAs are localized to the main vascular tissue (MVT), Aleurone layer (AL) and transfer cells (TCs; Fig. 8G–L). No signal has been detected in control seed sections hybridized with the corresponding sense probes, except for an unspecific signal found in the pericarp at W1 stage (Fig. 8M–O).

**Fig. 9:**
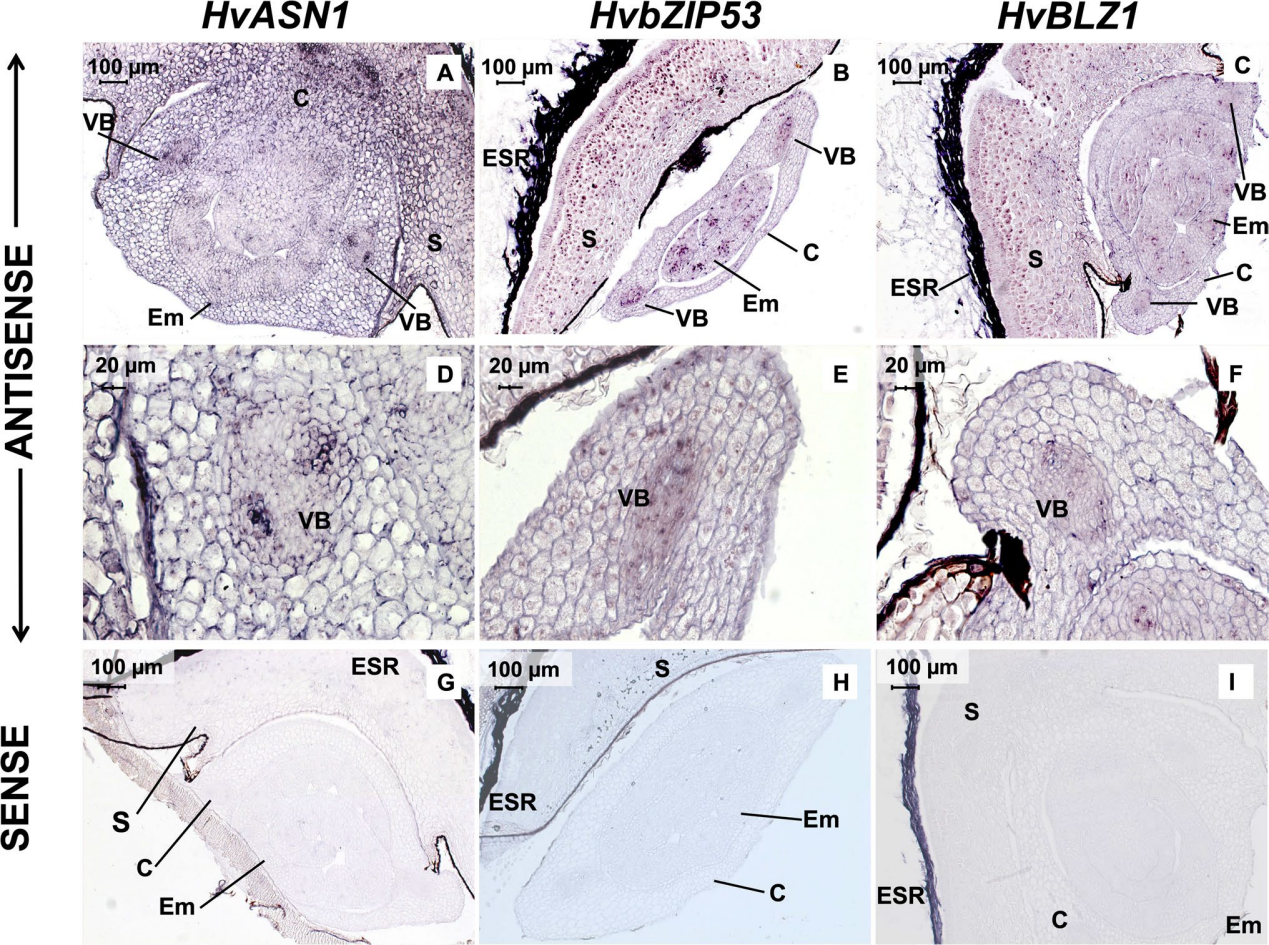
In situ mRNA hybridization analysis of *HvASN1, HvbZIP53*, and *HvBLZ1* in 30 h germinating *H. vulgare* seeds. **A**, **D**, **G** *HvASN1.* **B**, **E**, **H** *HvbZIP53.* **C**, **F**, **I** *HvBLZ1.* **A**–**C** Crosswise sections of germinating embryos. **D**, **E**, **F** Close-up of the vascular bundles in the coleoptile. **G**, **H**, **I** Control sense probes in germinating embryos. VB, vascular bundle; Em, embryo; S, scutellum, C, coleoptile; ESR, embryo surrounding region

Similarly, *mRNA *in situ hybridization assays have been performed at 30 hoi in germinating seeds to detect *HvASN1, HvbZIP53* and *HvBLZ1* co-localization (Fig. 9). The hybridized transversal sections of seeds showed strong signals for the transcripts of all three genes in the vascular bundles (VB), scutellum (S) and aleurone layer (AL; Fig. 9A–F). No signal has been found in control sections, except for an unspecific signal in the embryo surrounding region (ESR; Fig. 9G–I). Our results indicate that *HvASN1*, *HvbZIP53* and *HvBLZ1* exhibit a similar spatial expression pattern during seed development and germination, supporting the idea that HvASN1 transcription is regulated by both HvbZIP53 and HvBLZ1.

## Discussion

N supply plays a crucial role in plants, particularly under stress conditions when carbon availability is limited (Gaudinier et al. 2018). This fact is especially critical during energy-intensive physiological processes such as seed development and germination, which require significant N remobilization from low-photosynthetic tissues to actively growing ones (Hong et al. 2012). During germination, as nutrient reserves in the seed storage tissues are mobilized to support embryo development, N becomes essential for synthesizing amino acids, proteins, and other N-containing compounds needed for cellular metabolism (Coruzzi and Zhou 2001). Asparagine, the primary N recycling, transport and storage molecule, plays a key role in this process by facilitating N transport between tissues (Fujiki et al. 2001). This remobilization is particularly relevant in species like Arabidopsis, where N use efficiency during germination directly influences seedling growth, as well as in crops like barley, where it impacts overall yield (Gaufichon et al. 2010, 2013, 2017). Moreover, recent interest in the synthesis, accumulation, and degradation of asparagine in crops has grown due to its role as a precursor to acrylamide formation, a toxic compound generated during high-temperature cooking processes such as frying, baking, and roasting (Raffan and Halford 2021; Bachir et al. 2023).

ASN (EC 6.3.5.4) plays a crucial role in N transport and storage, as seen in *A. thaliana AtASN1 (DIN6)*, which is induced by darkness and repressed by carbon sources (Lam et al. 1994; Fujiki et al. 2001; Baena-González et al. 2007). Class-I genes, like *AtASN1*, are typically repressed by light or sugars, while Class-II genes, such as *AtASN2*, are not. This distinct regulation suggests that AtASN1 and AtASN2 may have different roles in N metabolism (Lam et al. 1998). In barley, five *ASN* family members have been identified (Møller et al. 2003; Avila-Ospina et al. 2015), with *HvASN1* and *HvASN2* clustering closely with *AtASN1* (Raffan and Halford 2021), suggesting a conserved role in N transport, particularly in seeds (Gaufichon et al. 2017). To explore the *HvASN1* role, we analyzed its expression in various organs and seed developmental stages, finding that it accumulates in flowers and embryos during seed maturation and germination coinciding with nutrient storage in the endosperm and nutrient mobilization. Our in situ hybridization experiments show that *HvASN1* transcripts localize in the embryo sac’s integuments during embryogenesis and at vascular tissues, aleurone layer, and transfer cells during embryo maturation and germination, supporting its function in nutrient transfer. Similarly, *AtASN1* transcripts and their corresponding proteins have been involved in N filling during Arabidopsis seed development and localized in the vascular tissues by in situ hybridization and immunolocalization assay, respectively (Alonso et al. 2009; Gaufichon et al. 2017). According to our results, upon imbibition, *HvASN1* expression peaks during germination, coinciding with endosperm remobilization, where starch and proteins are broken down into sugars and amino acids by hydrolytic enzymes from the scutellum and aleurone layer (Chrispeels and Varner 1967; Appleford and Lenton 1997). These findings support the role of *HvASN1* in N and nutrient remobilization, essential for seedling establishment. Other barley *ASN* genes, such as *HvASN3.2* (aka *HvASN4*) and *HvASN4* (aka *HvASN5*), are also dark-inducible (Møller et al. 2003; Avila-Ospina et al. 2015), indicating that multiple genes may contribute to N transport under carbon-limited conditions. The gene expression analyses show that *HvASN3* and *HvASN4* reach the highest levels in germinating barley and seedlings, while *HvASN2*, despite its high sequence identity (80.1%) with *HvASN1*, exhibits low expression, suggesting a specific localization likely in the grain as observed for *Triticum aestivum TaASN2* (Møller et al. 2003; International Barley Genome Sequencing Consortium 2012; Avila-Ospina et al. 2015; Gao et al. 2016; Raffan and Halford 2021). Whether HvASN1 and HvASN2 function redundantly or possess distinct biochemical and physiological roles remains unresolved. Our findings indicate that HvASN1 is pivotal for N transport and nutrient remobilization during seed development and germination in barley. However, based on evidence from wheat, it is plausible that HvASN2 fulfills a more specialized and functionally distinct role in seeds, underscoring the need for further studies to elucidate the functional divergence within the *ASN* gene family.

The regulation of N metabolism is a highly intricate process influenced by genetic, environmental, and hormonal factors (Gaudinier et al. 2018). The GCN family of TFs is one of those affected in the process. Thus, GCN4, a master transcriptional activator in the bZIP family, was initially characterized in *S. cerevisiae* for its role in responding to amino acid starvation (Hinnebusch and Natarajan 2002). This TF specifically binds to the promoter regions of genes involved in amino acid biosynthesis, coordinately inducing their expression (Arndt and Fink 1986). Interestingly, a conserved *cis*-regulatory element, known as the GCN-like motif or endosperm box, shares similarities with the binding sites for yeast GCN4 and mammalian bZIP TFs such as Jun and AP1 (Vogt et al. 1987). The GCN4 motif is found in multiple seed storage protein genes (e.g., rice glutelin; Wu et al. 1998) and helps regulate their seed-specific expression. In cereals like barley, the GCN-like motif is positioned approximately 300 bp upstream of the transcriptional start site of several seed storage protein genes and is typically adjacent to the endosperm motif (Kreis et al. 1985). These two elements form together the endosperm box, which is crucial for endosperm-specific gene expression and has been linked to N mobilization in seed storage proteins (Albani et al. 1997; Vicente-Carbajosa et al. 1998). The GCN-like motif is recognized by multiple bZIP TFs from different species, for example, barley BLZ1 and BLZ2, rice RISBZ1, REB, and RITA-1, maize Opaque-2, OHP1, OHP2, and wheat SPA and SHP (Pysh et al. 1993; Izawa et al. 1994; Albani et al. 1997; Nakase et al. 1997; Vicente-Carbajosa et al. 1998; Oñate et al. 1999; Onodera et al. 2001; Zhang et al. 2015; Boudet et al. 2019). The function of these TF in N metabolism, especially during seed development, highlights their relevance in coordinating nutrient storage and mobilization. Additionally, the bZIP TFs are important regulators in metabolic reprogramming and abiotic stress responses, which are essential for plant adaptation (Jakoby et al. 2002; Dröge-Laser et al. 2018; Lorenzo 2019). In this work, we present evidence that the GCN-like motif is implicated in the dark-induced expression of *HvASN1* in barley. This finding is consistent with observations in *A. thaliana*, where *AtASN1* upregulation in response to darkness is mediated by a G-box *cis*-element that binds bZIP-TFs, including AtbZIP53 (Fujiki et al. 2001; Baena-González et al. 2007; Alonso et al. 2009; Ávila-Ospina et al. 2015). Similarly, our results indicate that AtbZIP53 regulates *HvASN1* in barley, reinforcing the conserved role of these TFs in N metabolism across species.

The bZIP family comprises 78 members in Arabidopsis that play a crucial role in various developmental and stress-related processes. They are classified into 13 groups, with the S group being the largest (Dröge-Laser et al. 2018). The intricate interplay between bZIP TFs highlights their broad role in regulating nutrient allocation, mobilization, and stress tolerance in plants (Vicente-Carbajosa et al. 1998; Boudet et al. 2019). The coordinated regulation of N transport and mobilization at critical stages of seedling establishment emphasizes the importance of these TFs in maintaining efficient N use under fluctuating environmental conditions (Baena-González et al. 2007; Garg et al. 2019). Dimerization among bZIP TFs is critical for modulating DNA-binding specificity. S1 group bZIPs, such as bZIP53, preferentially form heterodimers with C group partners instead of homodimers, thereby expanding their regulatory potential (Garg et al. 2019; Li et al. 2023). Recent studies have demonstrated that these heterodimers can recognize novel DNA motifs not targeted by their monomeric forms, revealing a versatile regulatory network (Reinke et al. 2013; Li et al. 2023). For instance, in Arabidopsis, interactions among bZIP TFs such as GBF1, HY5, and HYH modulate photomorphogenesis and repress genes involved in seed germination (Ram and Chattopadhyay 2013; Iglesias-Fernández et al. 2014). AtbZIP53 enhances DNA-binding specificity and activates seed maturation and stress response genes through heterodimerization (Alonso et al. 2009). Although AtbZIP53 does not directly interact with ABI3, it likely forms a ternary complex with AtbZIP10, AtbZIP25, and AtABI3 to regulate seed maturation (Alonso et al. 2009). In this context, we explored the potential heterodimerization between HvbZIP53 and HvBLZ1, a C group bZIP TF known to enhance grain storage protein transcription by binding to a GCN-like element (Vicente-Carbajosa et al. 1998). While both HvbZIP53 and HvBLZ1 are expressed in various plant organs, their temporal expression patterns in developing and germinating embryos closely resemble those of HvASN1, suggesting that these two bZIPs may act as upstream regulators of HvASN1 during the seed life cycle. In the present study, we demonstrate that HvBLZ1 interacts with HvbZIP53 in Y2H assays, broadening the functional role of these TFs in nutrient allocation and stress adaptation. Similarly, we observed transactivation of *HvASN1* promoter by bZIP53-BLZ1 complex in an Arabidopsis system, providing direct evidence of this interaction and its regulatory effect on *HvASN1* gene expression. Nevertheless, the early induction of *HvASN1* in the endosperm during the initial stages of imbibition, compared to *HvbZIP53* and *H*vBLZ1, may suggest the involvement of transcriptional regulators that are specific to the endosperm and act independently of HvbZIP53 and HvBLZ1. In addition, the relatively low levels of *HvASN1* transcripts detected in the endosperm imply that its role in nitrogen remobilization within this tissue may be limited or conditional, in contrast to its more prominent function in the embryo. Overall, our results align with previous studies showing that bZIP53, along with other members of the C/S1 network, regulates *ASN1* (Baena-González et al. 2007; Hanson et al. 2008; Alonso et al. 2009). Amino acid synthesis, transport, and seed storage protein synthesis are tightly linked processes that must be coordinated in both vegetative tissues and seeds. In line with this, ectopic overexpression of *AtASN1* has been shown to enhance seed storage protein content and improve the N status of the seed in *A. thaliana* (Lam et al. 2003). These findings highlight the importance of bZIP53 in *ASN1* regulation and its broader role in seed N metabolism.

Sugars play a critical function in regulating the expression of bZIP TFs (Wang et al. 2022). Similar to the regulation of Arabidopsis S group of bZIPs like AtbZIP11 and AtbZIP53 (Weltmeier et al. 2009), we found that the HvbZIP53 mRNA contains a conserved uORF in the 5' UTR, which regulates its post-translational synthesis. Using the *NAN* marker gene as a reporter, we demonstrated in this study (Fig. 5) that sucrose represses HvbZIP53 translation via the uORF. This sucrose-induced repression of translation follows a mechanism similar to that observed in AtbZIP11 (Wiese et al. 2004). The conserved uORF in HvbZIP53 suggests that its sucrose regulation is integrated into a broader metabolic signaling pathway that governs bZIP TFs in barley, especially in response to environmental and metabolic signals like light, darkness, and sucrose. This post-translational regulatory mechanism provides greater flexibility and allows for a faster response when compared to transcriptional regulation. The high conservation of uORF leader sequences in S1 bZIPs strengthens the possibility that the sucrose-induced repression of translation mechanism is widely conserved across the plant kingdom (Wiese et al. 2004). The results of Wiese et al. (2004), along with those presented in the present work, emphasize the complex interactions between various regulatory mechanisms that govern the expression of bZIP TFs (Wetlmeier et al. 2009).

## Conclusion

This study underscores the crucial role of N transport and storage through asparagine metabolism during seed development and germination in barley, a key cereal crop. By identifying a *GCN*-like motif in the *HvASN1* promoter and demonstrating its interaction with bZIP TFs like bZIP53 and BLZ1, we gain important insights into the complex regulation of N use efficiency. This regulation is not only responsive to environmental signals like light/darkness and stress but also plays a key role throughout seed development. The ability of bZIP53 to regulate N-related genes across various species, together with its interaction with C group bZIPs like BLZ1, suggests a conserved mechanism that integrates nutrient availability with seed developmental processes. Our results also demonstrate that bZIP53 is involved in the regulation of N metabolic genes in response to sucrose, where a post-translational mechanism, resembling the sucrose-induced repression of translation observed in Arabidopsis, governs its protein abundance. This last result highlights the interplay between sugar signaling and N metabolism. Manipulating the expression of *HvASN1* and *HvbZIP53* presents a promising strategy for enhancing nitrogen use efficiency (NUE) in barley. Evidence from other cereals supports this idea: in maize, the teosinte-derived *THP9* allele was shown to increase free amino acid content and seed protein levels (Huang et al. 2022), while in rice overexpression of *OsASN1* improved grain nitrogen and protein content under low-nitrogen conditions (Lee et al. 2020). Likewise, the modification of bZIP transcription factors in wheat, such as TabZIP60, has been associated with improved NUE and increased grain yield (Yang et al. 2019). These findings suggest that targeted manipulation of *HvASN1* and *HvbZIP53* could be a promising strategy for breeding barley varieties with improved nitrogen efficiency and grain quality.

## Supplementary Information

Below is the link to the electronic supplementary material.

Supplementary Fig. S1 In silico analysis of Hordeum vulgare asparagine synthetase 1 and 2 (HvASN1, HvASN2). Phylogenetic tree (**A**) and schematic distribution of conserved amino acid motifs (**B**) of ASN1 and ASN2 sequences in Triticeae. Bootstrapping values are shown on the tree branches. The position of the glutamine amidotransferase (GATase) and the asparagine synthetase ASN active domains are indicated (EXPASY Database; https://prosite.expasy.org). *Aet*, *Aegilops tauschii*; *Hv*, *Hordeum vulgare*; *Ta*, *Triticum aestivum*; *Tu*, *Triticum urartu*; *At*, *Arabidopsis thaliana* file1 (PNG 80 KB)

Supplementary Fig. S2 Negative control of transient expression assays. The infiltration of p19 and pGWBZ empty vector into *N. benthamiana* leaves do not produce an increase of luciferase activity file2 (PNG 393 KB)

Supplementary Fig. S3 Phylogenetic dendrogram and schematic distribution of the conserved motifs found in the sequences of bZIP53 and putative orthologous bZIP TFs in some members of the Poaceae family and *A. thaliana* studied in this work. **A** Sequences of the conserved amino acid motifs. **B** Bootstrapping values are shown on the branches of the tree. Nuclear localization signal in motif 1 is marked with an asterisk in A and its sequence is shown in bold in **B** file3 (PNG 560 KB)

Supplementary Fig. S4 Transactivation assays using HvbZIP53 and HvBLZ1 TF under the transcriptional control of the CaMV 35S as effectors. The *HvASN1* gene promoter driving the expression of the *uidA* gene (GUS activity) has been used as reporter. **A** Schematic representation of the effector and reporter constructs used in the assay. **B** NAN activity has been evaluated after co-bombardment of the effector and reporter combinations indicated in **A** on *A. thaliana* leaves. The GUS activity has been used to standardize the variations in the efficiency of the transformation. The relative amounts of reporter and effector plasmids used in these assays correspond to a 1:1 ratio. Values are means ± SE of four independent replicates file4 (PNG 301 KB)

Supplementary Fig. S5 Expression analysis of *HvASN1*, *HvbZIP53*, and *HvBLZ1* genes by qPCR in different barley tissues. Data are means ± SE of two technical replicates of three biological samples file5 (PNG 501 KB)

Supplementary Fig. S6 Profile of qPCR Ct values of *HvGAPDH* gene throughout barley seed development and germination. Data are means ± SE of two technical replicates of three biological samples file6 (PNG 227 KB)

Supplementary Table S1 Locus of asparagine synthetase enzymes and bZIP TFs used in the analysis file7 (PNG 154 KB)

Supplementary Table S2 Consensus sequences of motifs obtained using MEME tool in Hv, Ta, Aet, and TuASN1/2 proteins and their E-values file8 (JPG 447 KB)

Supplementary Table S3 Promoter sequences of ASN genes of H. vulgare (cv. Bomi and ssp. *vulgare* L.), *T. aestivum*, *T. urartu*, and *A. tauschii* used for cloning and in the phylogenetic shadowing analysis shown in Fig. 1 (Green: GCN-like element) file9 (PNG 95 KB)

Supplementary Table S4 List of primers used file10 (PNG 190 KB)

## Data Availability

All the data used in this work are included in the manuscript.

## Abbreviations

ASN: Asparagine synthetase
CDS: Coding sequence
GUS: Glucuronidase
hoi: Hours of imbibition
LUC: Luciferase
NAN: Neuraminidase
TF: Transcription factor
uORF: Upstream open reading frame
Y2H: Yeast two-hybrid
