# Supplementary figures and images for: Regulation of HvASN1 expression by bZIP transcription factors during barley embryo development and germination
Source: Planta. 2025 Jun 9;262(1):20. doi: 10.1007/s00425-025-04730-0 (PMC12148973; doi:10.1007/s00425-025-04730-0)

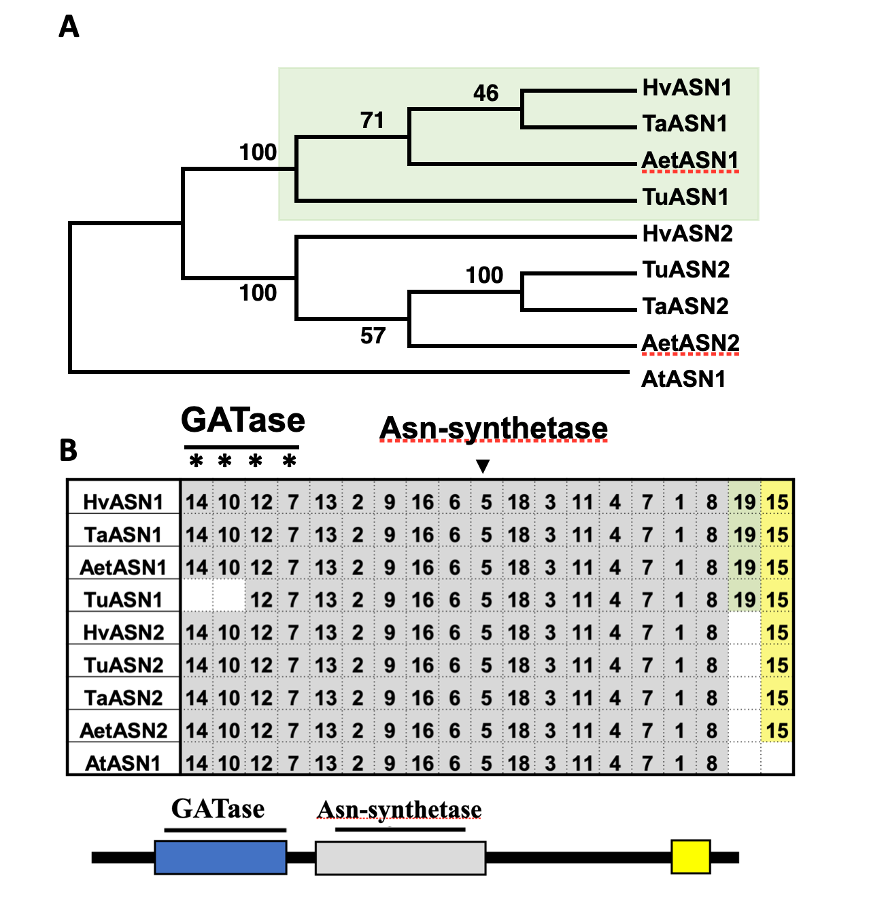

Supplement: Supplementary file 1 — Supplementary Fig. S1 In silico analysis of Hordeum vulgare asparagine synthetase 1 and 2 (HvASN1, HvASN2). Phylogenetic tree (A) and schematic distribution of conserved amino acid motifs (B) of ASN1 and ASN2 sequences in Triticeae. Bootstrapping values are shown on the tree branches. The position of the glutamine amidotransferase (GATase) and the asparagine synthetase ASN active domains are indicated (EXPASY Database; https://prosite.expasy.org). Aet, Aegilops tauschii; Hv, Hordeum vulgare; Ta, Triticum aestivum; Tu, Triticum urartu; At, Arabidopsis thaliana file1 (PNG 80 KB) [file 425_2025_4730_MOESM1_ESM.png]

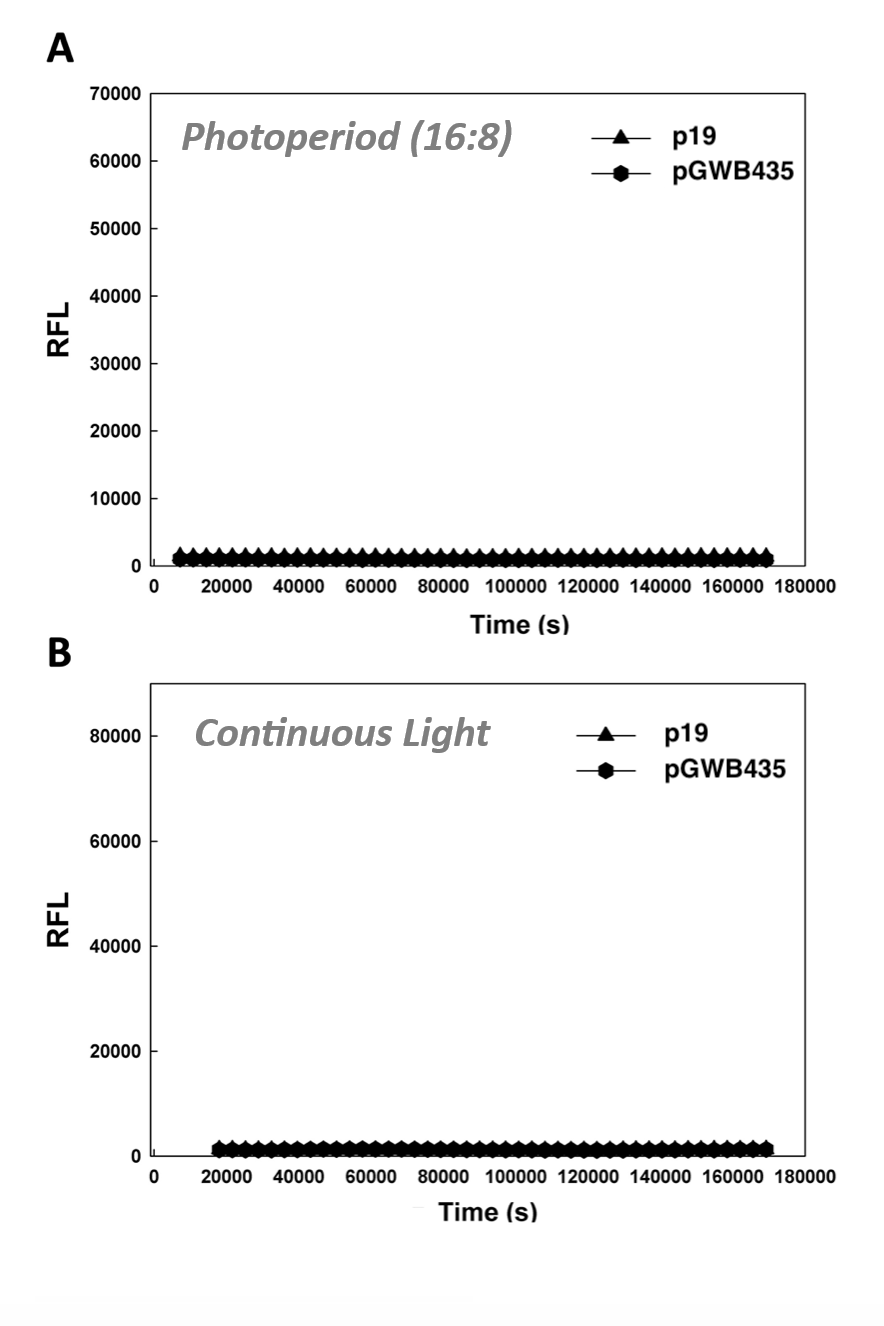

Supplement: Supplementary file 2 — Supplementary Fig. S2 Negative control of transient expression assays. The infiltration of p19 and pGWBZ empty vector into N. benthamiana leaves do not produce an increase of luciferase activity file2 (PNG 393 KB) [file 425_2025_4730_MOESM2_ESM.png]

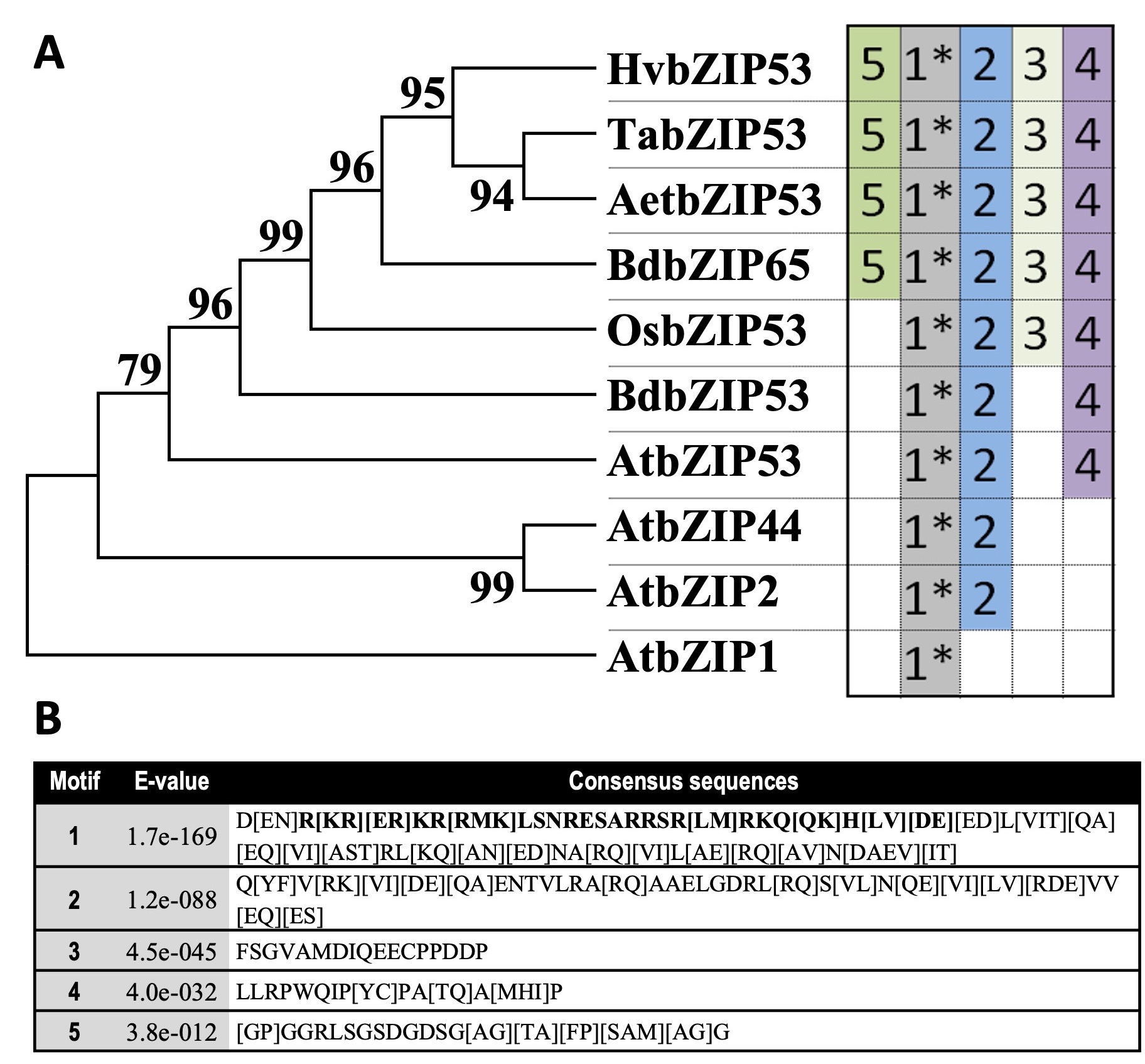

Supplement: Supplementary file 3 — Supplementary Fig. S3 Phylogenetic dendrogram and schematic distribution of the conserved motifs found in the sequences of bZIP53 and putative orthologous bZIP TFs in some members of the Poaceae family and A. thaliana studied in this work. A Sequences of the conserved amino acid motifs. B Bootstrapping values are shown on the branches of the tree. Nuclear localization signal in motif 1 is marked with an asterisk in A and its sequence is shown in bold in B file3 (PNG 560 KB) [file 425_2025_4730_MOESM3_ESM.jpg]

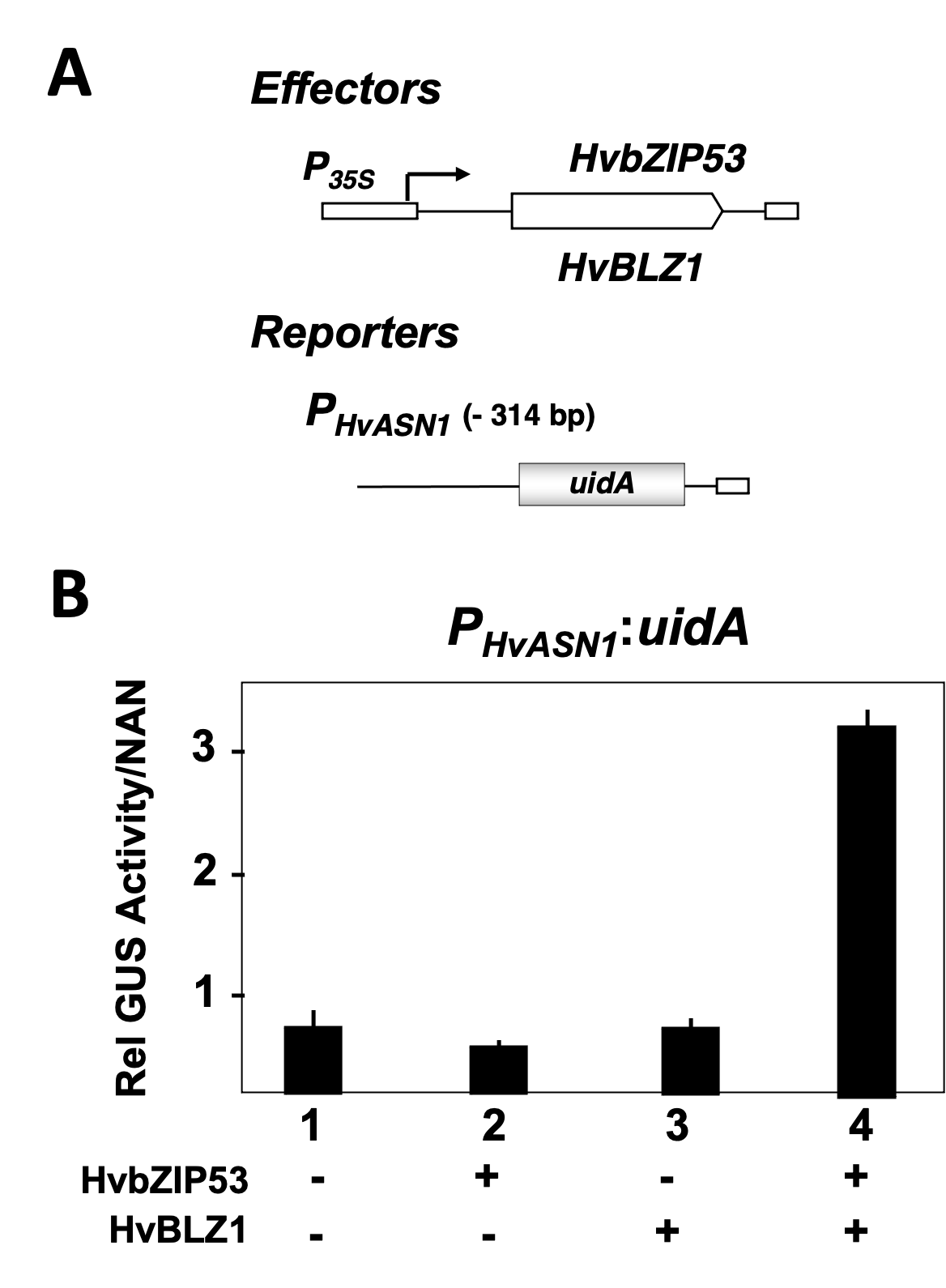

Supplement: Supplementary file 4 — Supplementary Fig. S4 Transactivation assays using HvbZIP53 and HvBLZ1 TF under the transcriptional control of the CaMV 35S as effectors. The HvASN1 gene promoter driving the expression of the uidA gene (GUS activity) has been used as reporter. A Schematic representation of the effector and reporter constructs used in the assay. B NAN activity has been evaluated after co-bombardment of the effector and reporter combinations indicated in A on A. thaliana leaves. The GUS activity has been used to standardize the variations in the efficiency of the transformation. The relative amounts of reporter and effector plasmids used in these assays correspond to a 1:1 ratio. Values are means ± SE of four independent replicates file4 (PNG 301 KB) [file 425_2025_4730_MOESM4_ESM.png]

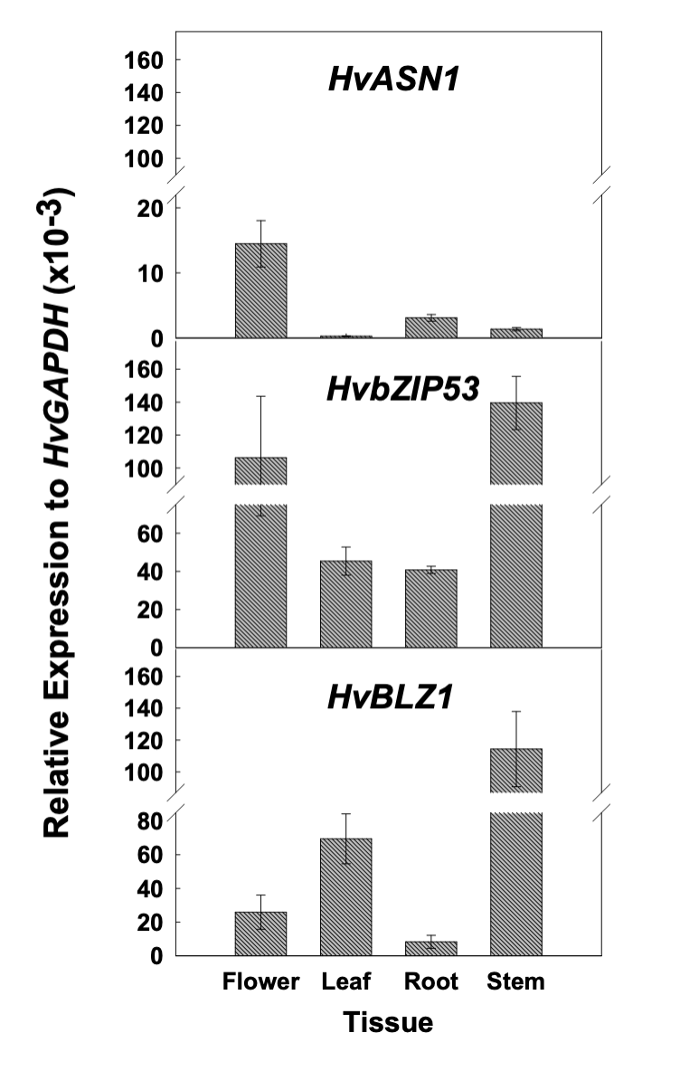

Supplement: Supplementary file 5 — Supplementary Fig. S5 Expression analysis of HvASN1, HvbZIP53, and HvBLZ1 genes by qPCR in different barley tissues. Data are means ± SE of two technical replicates of three biological samples file5 (PNG 501 KB) [file 425_2025_4730_MOESM5_ESM.png]

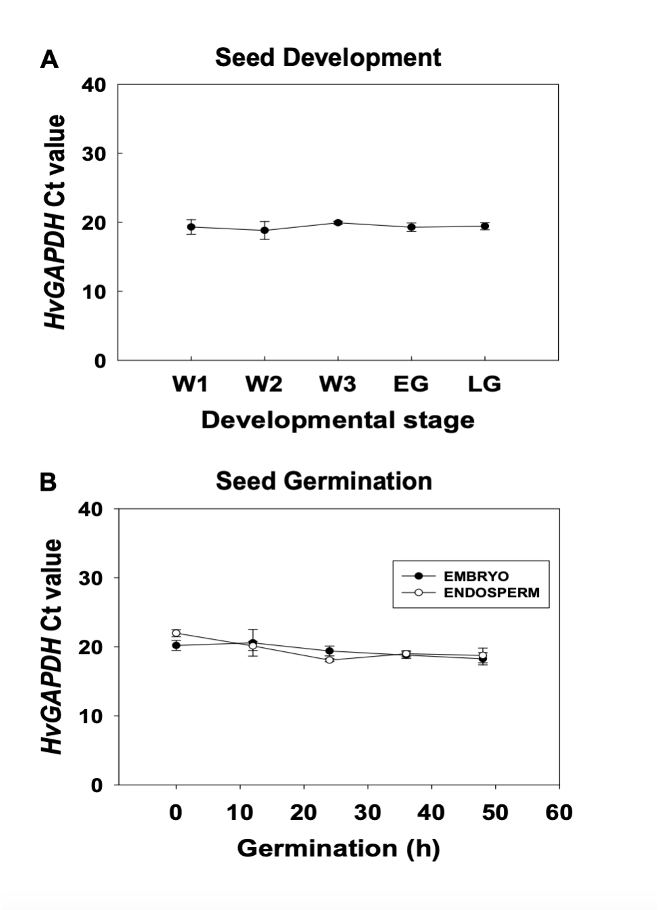

Supplement: Supplementary file 6 — Supplementary Fig. S6 Profile of qPCR Ct values of HvGAPDH gene throughout barley seed development and germination. Data are means ± SE of two technical replicates of three biological samples file6 (PNG 227 KB) [file 425_2025_4730_MOESM6_ESM.png]

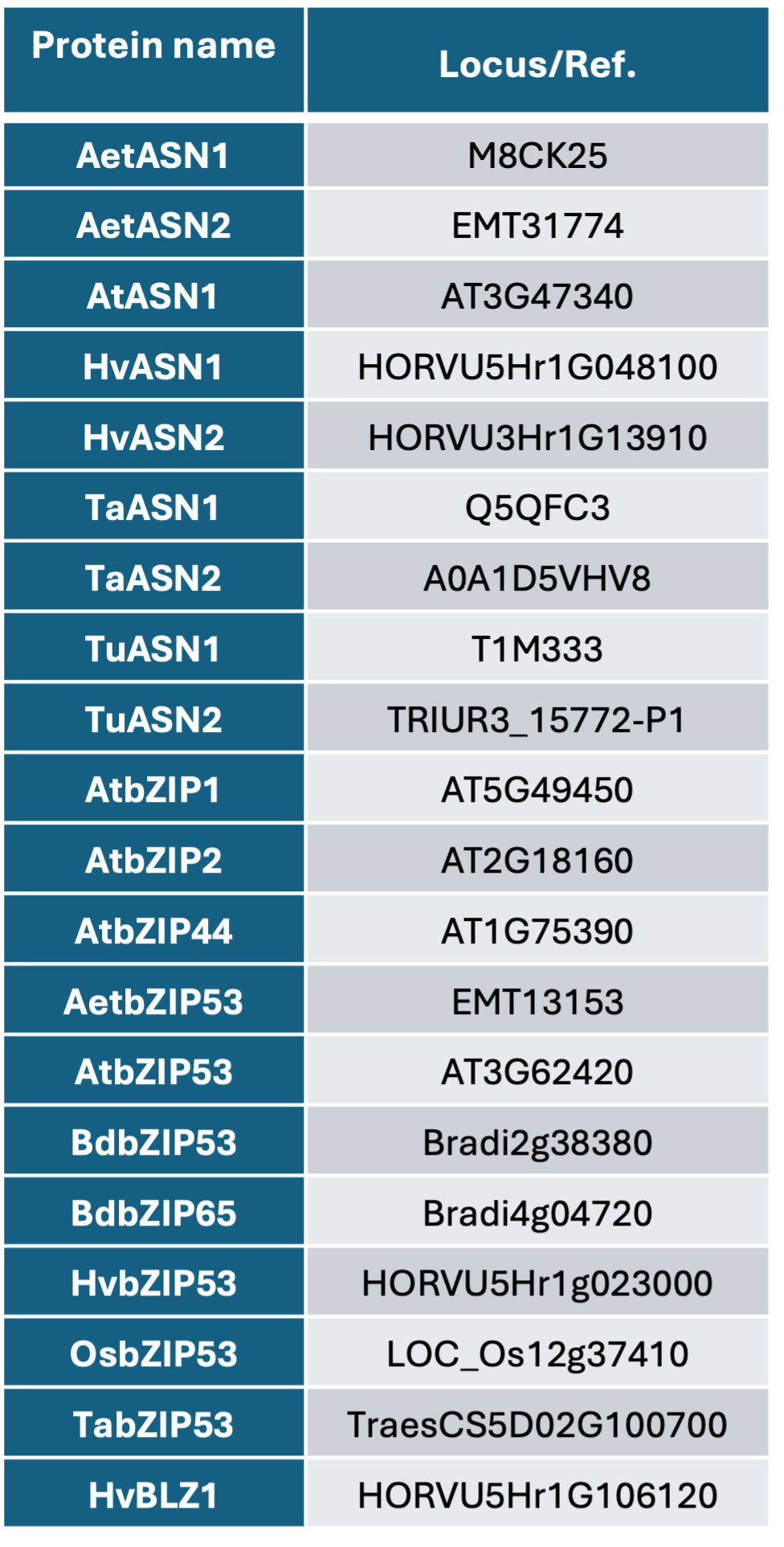

Supplement: Supplementary file 7 — Supplementary Table S1 Locus of asparagine synthetase enzymes and bZIP TFs used in the analysis file7 (PNG 154 KB) [file 425_2025_4730_MOESM7_ESM.png]

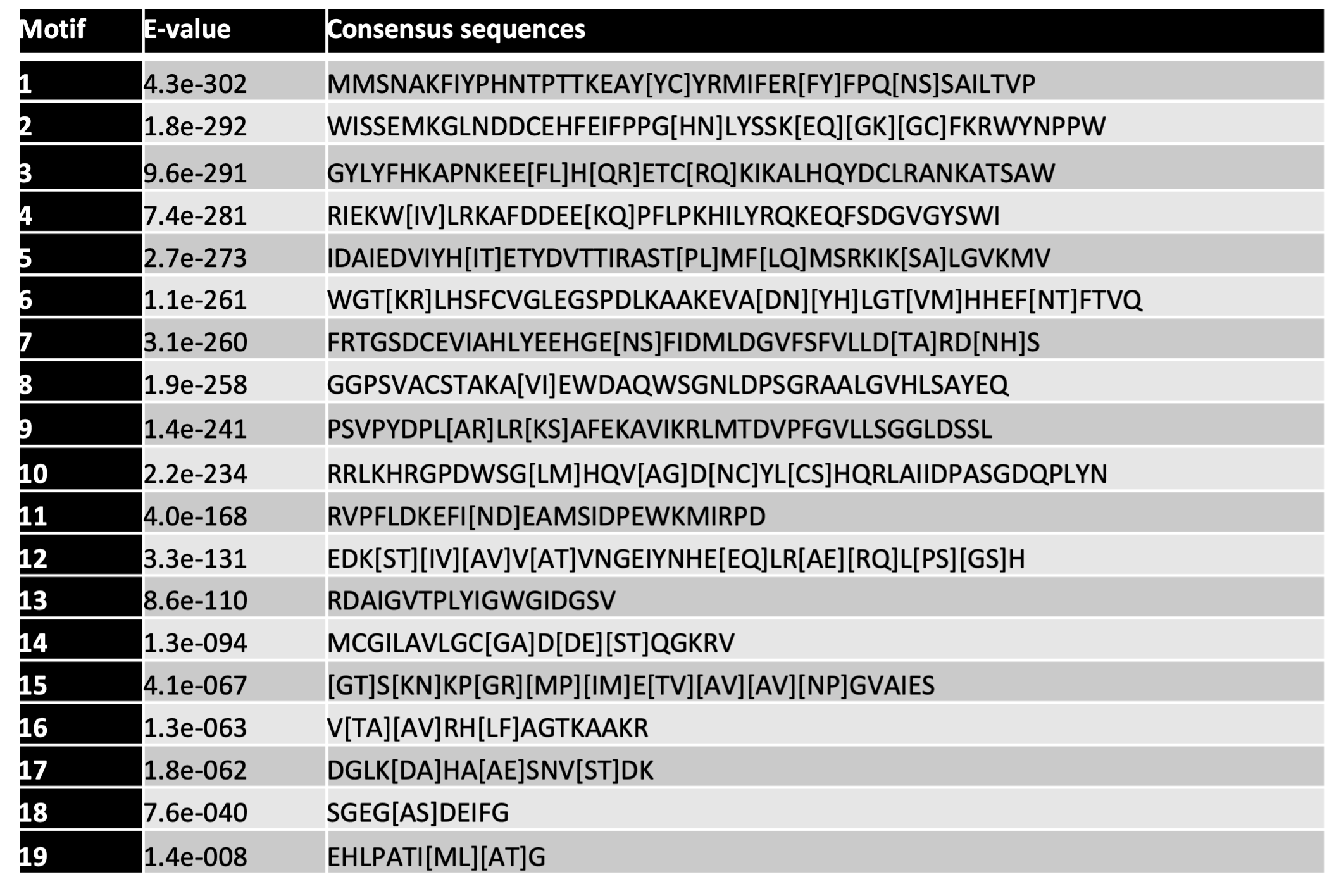

Supplement: Supplementary file 8 — Supplementary Table S2 Consensus sequences of motifs obtained using MEME tool in Hv, Ta, Aet, and TuASN1/2 proteins and their E-values file8 (JPG 447 KB) [file 425_2025_4730_MOESM8_ESM.png]

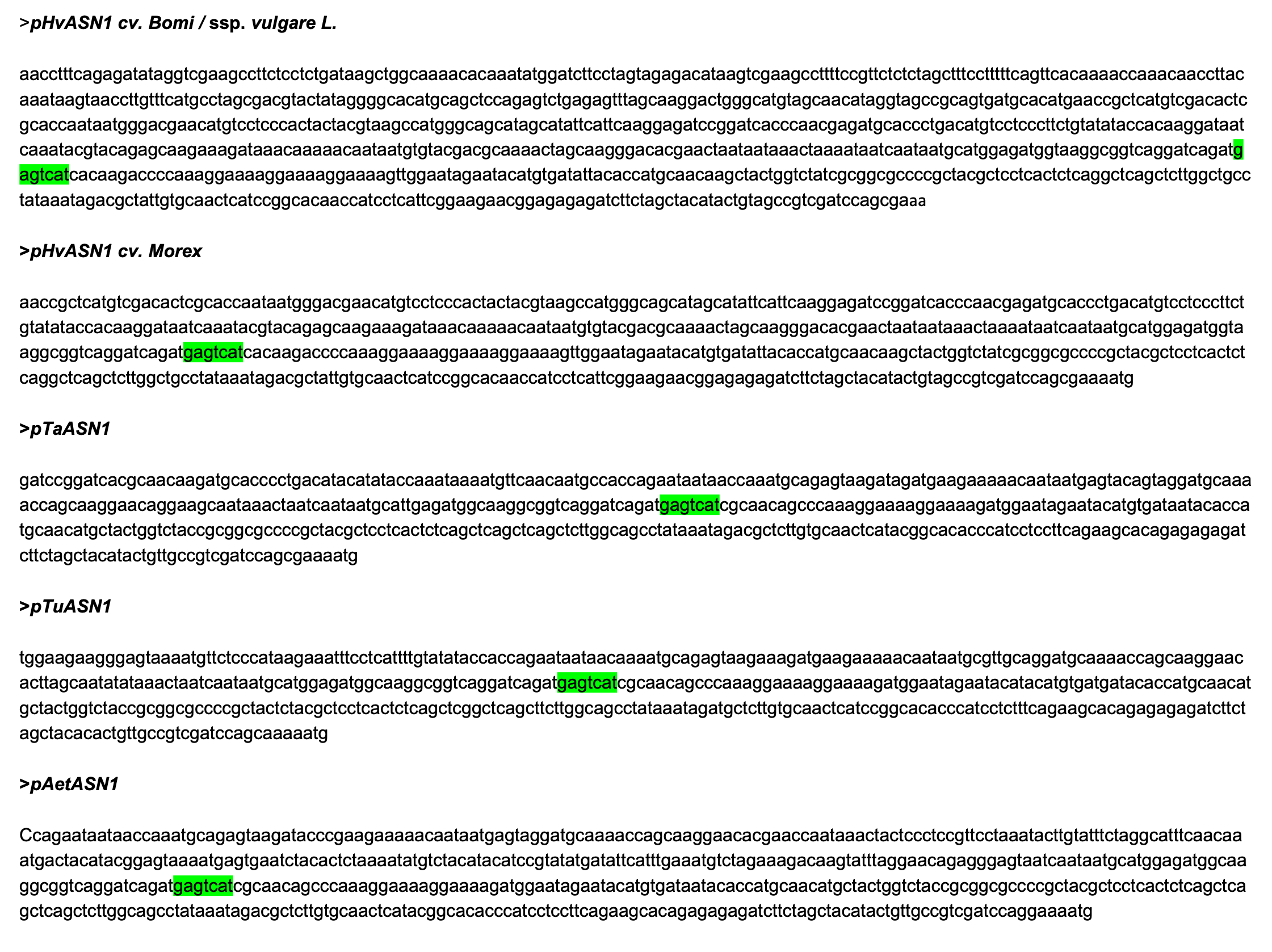

Supplement: Supplementary file 9 — Supplementary Table S3 Promoter sequences of ASN genes of H. vulgare (cv. Bomi and ssp. vulgare L.), T. aestivum, T. urartu, and A. tauschii used for cloning and in the phylogenetic shadowing analysis shown in Fig. 1 (Green: GCN-like element) file9 (PNG 95 KB) [file 425_2025_4730_MOESM9_ESM.png]

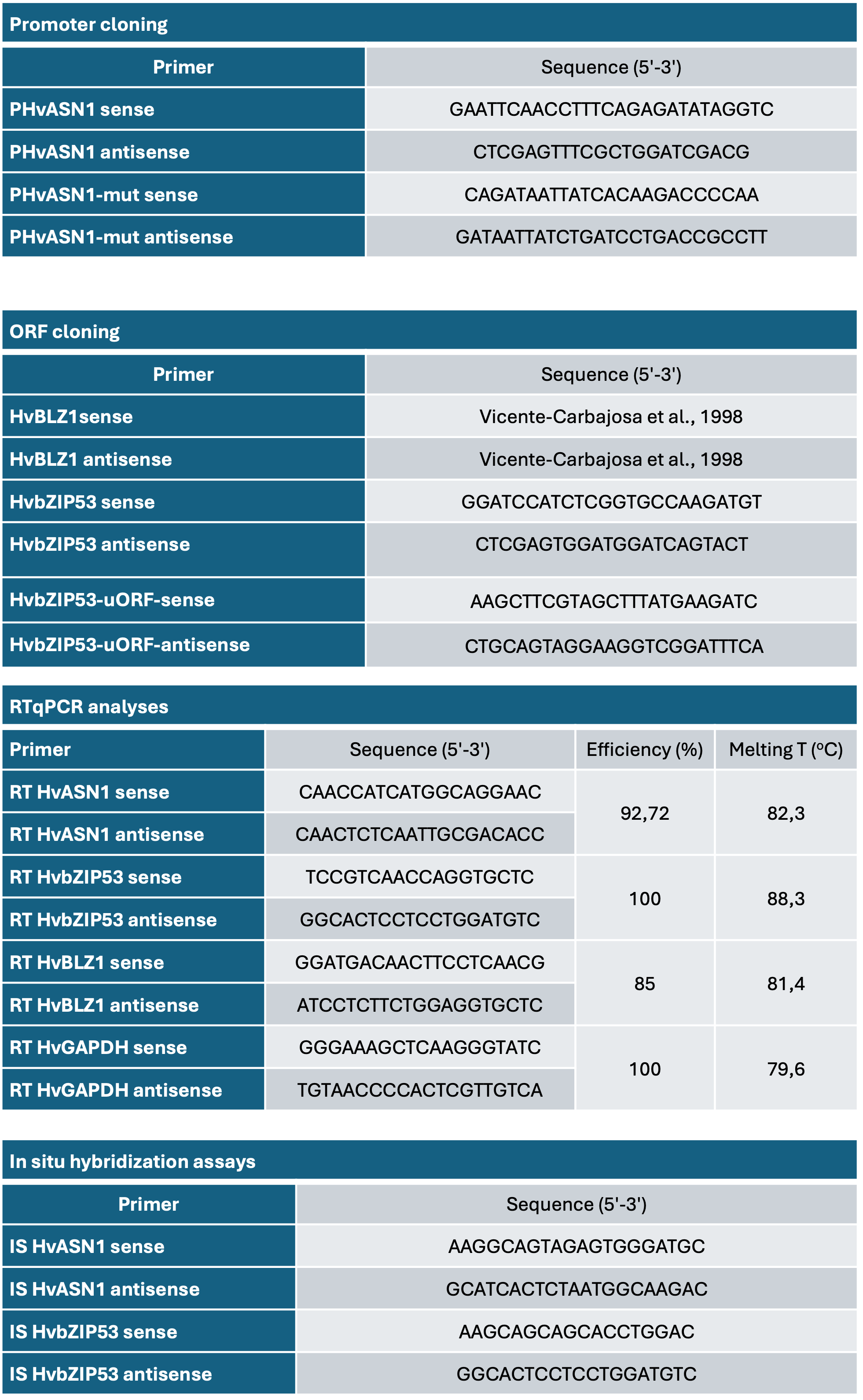

Supplement: Supplementary file 10 — Supplementary Table S4 List of primers used file10 (PNG 190 KB) [file 425_2025_4730_MOESM10_ESM.png]
